# Supplementary material for: Phylogenomic analysis of target enrichment and transcriptome data uncovers rapid radiation and extensive hybridization in the slipper orchid genus Cypripedium
Source: Ann Bot. 2024 Sep 12;134(7):1229–50. doi: 10.1093/aob/mcae161 (PMC11688532; doi:10.1093/aob/mcae161)
Supplement: mcae161_suppl_Supplementary_Figure_S1 [file mcae161_suppl_supplementary_figure_s1.docx]

Figure S1: Distribution of *Cypripedium* species per section [classification following Frosch and Cribb (2012); distribution information based on Eccarius (2009), Frosch and Cribb (2012), Chen *et al.* (2013), and Walid et al. (2019)].


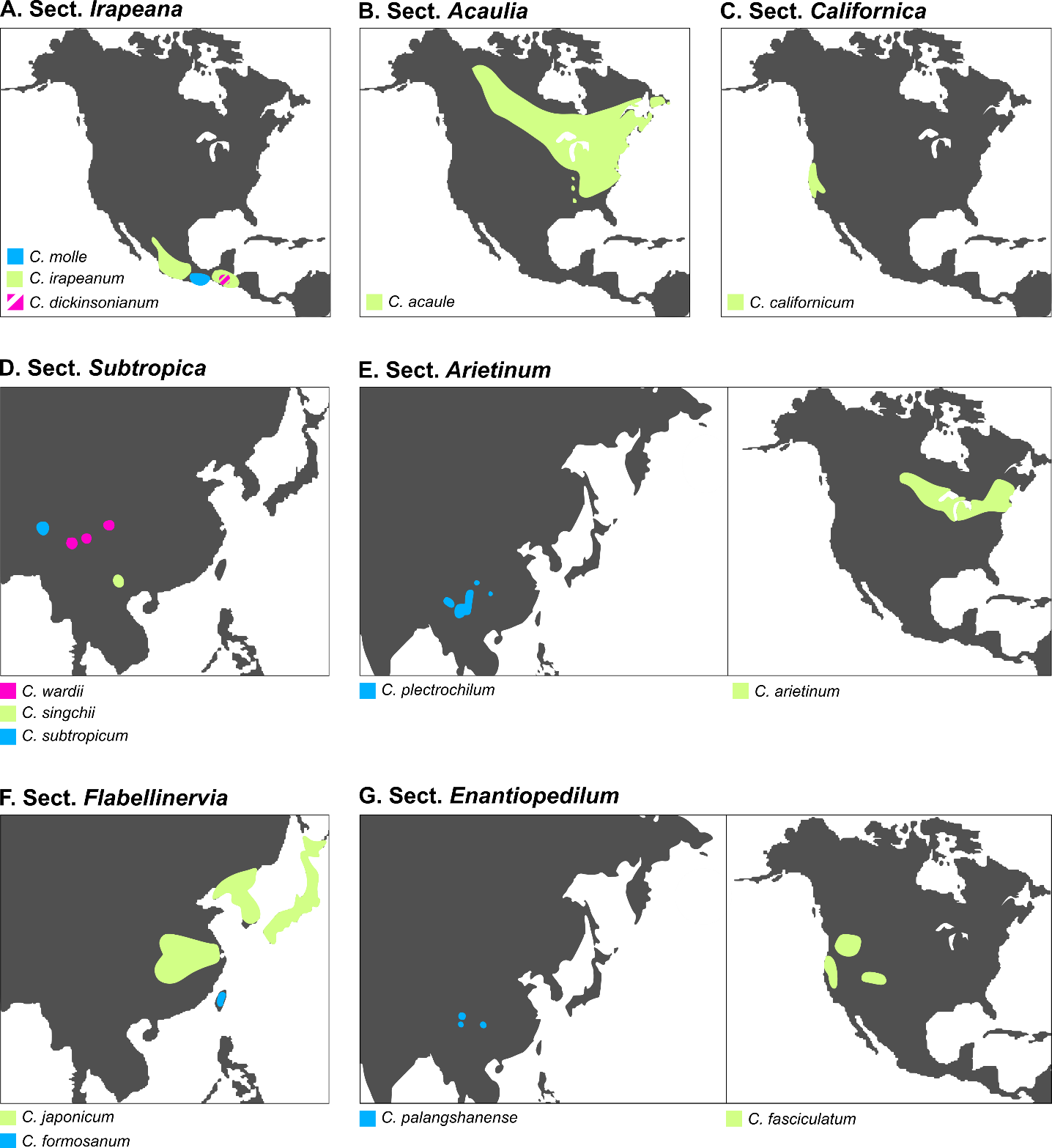


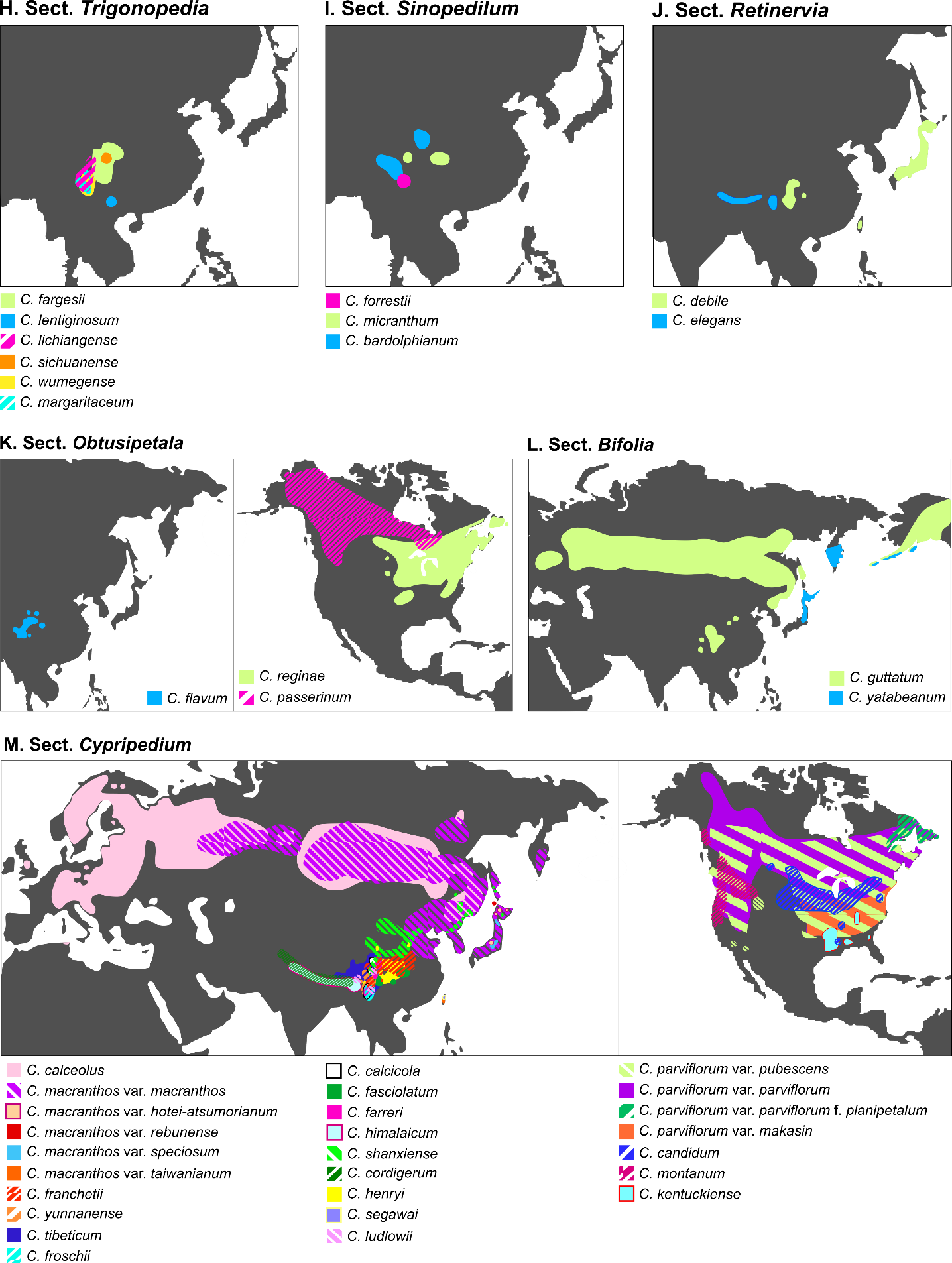


LITERATURE CITED

**Chen SC, Liu ZJ, Chen LJ, Li LQ**. **2013**. *The Genus Cypripedium in China*. Peking: Science Press.

**Eccarius W**. **2009**. *Orchideengattung Cypripedium*. EchinoMedia.

**Frosch W, Cribb P**. **2012**. *Hardy Cypripedium: Species, hybrids and cultivation*. Kew Publishing Kew.

**Walid N, Rebbas K, Krouchi F**. **2019**. Découverte de Cypripedium calceolus (Orchidaceae) au Djurdjura (Algérie), nouvelle pour l’Afrique du Nord. *Flora Mediterranea* **29**: 207–214.
