# Supplementary material for: Phylogenomic analysis of target enrichment and transcriptome data uncovers rapid radiation and extensive hybridization in the slipper orchid genus Cypripedium
Source: Ann Bot. 2024 Sep 12;134(7):1229–50. doi: 10.1093/aob/mcae161 (PMC11688532; doi:10.1093/aob/mcae161)
Supplement: mcae161_suppl_Supplementary_Tables [file mcae161_suppl_supplementary_tables.docx]

Table S1: Selected examples of taxonomic revisions for the infrageneric classification of *Cypripedium*.

| Lindley 1840 | Pfitzer 1903 | Cribb 1997 | Eccarius 2009 | Frosch & Cribb 2012 | Chen *et al.* 2013 |
| --- | --- | --- | --- | --- | --- |
|  |  | **Sect. *Subtropica*** | **Sect. *Subtropica*** | **Sect. *Subtropica*** | **Sect. *Subtropica*** |
|  |  | *C. subtropicum* | *C. subtropicum* | *C. subtropicum* | *C. subtropicum* |
|  |  |  |  |  | *C. singchii* |
|  |  |  |  |  | **Sect. *Wardiana*** |
|  |  | *C. wardii* | *C. wardii* | *C. wardii* | *C. wardii* |
| ***Foliosa* group** (lateral sepals free at apex) | **Series *Arcuinervia* Sect. *Eucypripedium* Subsect. *Obtusipetala*** | **Sect. *Irapeana*** | **Sect. *Irapeana*** | **Sect. *Irapeana*** | **Sect. *Irapeana*** |
| *C. irapeanum* | *C. irapeanum* | *C. irapeanum* | *C. irapeanum* | *C. irapeanum* | *C. irapeanum* |
| *C. molle* |  | *C. molle* | *C. mole* (as *C. irapeanum* ssp. *molle*) | *C. molle* | *C. molle* |
|  |  | *C. dickinsonianum* | *C. dickinsonianum* | *C. dickinsonianum* | *C. dickinsonianum* |
|  |  |  |  | **Sect. *Californica*** | **Sect. *Californica*** |
|  | *C. californicum* | *C. californicum* | *C. californicum* | *C. californicum* | *C. californicum* |
|  | **Series *Arcuinervia* Sect. *Eucypripedium* Subsect. *Acutipetala*** | **Sect. *Cypripedium* Subsect. *Cypripedium*** | **Sect. *Cypripedium*** | **Sect. *Cypripedium* Subsect. *Cypripedium*** | **Sect. *Cypripedium* Subsect. *Cypripedium*** |
| *C. calceolus* | *C. calceolus* | *C. calceolus* | *C. calceolus* | *C. calceolus* | *C. calceolus* |
|  | *C. henryi* | *C. henryi* | *C. henryi* | *C. henryi* | *C. henryi* |
|  |  | *C. shanxiense* | *C. shanxiense* | *C. shanxiense* | *C. shanxiense* |
|  |  | *C. segawai* | *C. segawai* | *C. segawai* | *C. segawae* |
| *C. cordigerum* | *C. cordigerum* | *C. cordigerum* | *C. cordigerum* | *C. cordigerum* | *C. cordigerum* |
| *C. parviflorum* (incl. *C. pubescens*) | *C. parviflorum* (incl. *C. pubescens*) | *C. parviflorum* (incl. *C. pubescens*) | *C. parviflorum* (incl. *C. pubescens*) | *C. parviflorum* (incl. *C. pubescens*) | *C. parviflorum* (incl. *C. pubescens*) |
| *C. candidum* | *C. candidum* | *C. candidum* | *C. candidum* | *C. candidum* | *C. candidum* |
| *C. montanum* | *C. montanum* | *C. montanum* | *C. montanum* | *C. montanum* | *C. montanum* |
|  |  |  | *C. kentuckiense* | *C. kentuckiense* | *C. kentuckiense* |
|  | *C. fasciolatum* | *C. fasciolatum* | *C. fasciolatum* | *C. fasciolatum* |  |
|  |  | *C. farreri* | *C. farreri* (as *C. fasciolatum* ssp. *farreri*) | *C. farreri* |  |
|  |  | **Sect. *Cypripedium* Subsect. *Macrantha*** | **Sect. *Macrantha*** | **Sect. *Cypripedium* Subsect. *Macrantha*** | **Sect. *Cypripedium* Subsect. *Macrantha*** |
|  |  |  |  |  | *C. fasciolatum* |
|  |  |  |  |  | *C. farreri* |
|  | *C. himalaicum* | *C. himalaicum* | *C. himalaicum* | *C. himalaicum* | *C. himalaicum* |
| *C. macranthos* | *C. macranthos* (incl. *C. thunbergii*) | *C. macranthos* | *C. macranthum* | *C. macranthos* | *C. macranthos* |
|  |  | *C. tibeticum* | *C. tibeticum* | *C. tibeticum* | *C. tibeticum* |
|  | *C. corrugatum* | *C. corrugatum* |  |  |  |
|  |  | *C. calcicola* (as *C. smithii*) | *C. calcicola* (as *C. tibeticum* ssp. *calcicola*) | *C. calcicola* | *C. calcicola* |
|  | *C. yunnanense* | *C. yunnanense* | *C. yunnanense* | *C. yunnanense* | *C. yunnanense* |
|  |  | *C. ludlowii* | *C. ludlowii* (as *C. tibeticum* ssp. *ludlowii*) | *C. ludlowii* | *C. ludlowii* |
|  |  | *C. franchetii* | *C. franchetii* | *C. franchetii* | *C. franchetii* |
|  |  |  | *C. froschii* (as *C. tibeticum* var. *froschii*) | *C. froschii* |  |
|  |  |  |  |  | *C. taibaiense* |
|  | **Series *Arcuinervia* Sect. *Enantiopedilum*** | **Sect. *Enantiopedilum*** | **Sect. *Enantiopedilum*** | **Sect. *Enantiopedilum*** | **Sect. *Enantiopedilum*** |
|  | *C. fasciculatum* | *C. fasciculatum* | *C. fasciculatum* | *C. fasciculatum* | *C. fasciculatum* |
|  |  |  |  | *C. palangshanense* |  |
| ***Foliosa group*** (lateral sepals connate to the apex) | **Series *Arcuinervia* Sect. *Eucypripedium* Subsect. *Obtusipetala*** | **Sect. *Obtusipetala*** | **Sect. *Obtusiflora*** | **Sect. *Obtusipetala*** | **Sect. *Obtusipetala*** |
|  | *C. flavum* (as *C. luteum*) | *C. flavum* | *C. flavum* | *C. flavum* | *C. flavum* |
| *C. reginae* (as *C. spectabile*) | *C. reginae* | *C. reginae* | *C. reginae* | *C. reginae* | *C. reginae* |
| *C. passerinum* | *C. passerinum* | *C. passerinum* | *C. passerinum* | *C. passerinum* | *C. passerinum* |
| ***Acaulia* group** | **Series *Arcuinervia* Sect. *Eucypripedium* Subsect. *Acutipetala*** | **Sect. *Acaulia*** | **Sect. *Acaulia*** | **Sect. *Acaulia*** | **Sect. *Acaulia*** |
| *C. acaule* (as *C. humile*) | *C. acaule* | *C. acaule* | *C. acaule* | *C. acaule* | *C. acaule* |
|  | **Sect. *Retinervia*** | **Sect. *Retinervia*** | **Sect. *Retinervia*** | **Sect. *Retinervia*** | **Sect. *Retinervia*** |
|  |  | *C. elegans* | *C. elegans* | *C. elegans* | *C. elegans* |
|  | *C. debile* | *C. debile* | *C. debile* | *C. debile* | *C. debile* |
|  |  |  |  |  | **Sect. *Palangshanensia*** |
|  |  | *C. palangshanense* | *C. palangshanense* |  | *C. palangshanense* |
| ***Bifolia* group** | **Series *Arcuinervia* Sect. *Eucypripedium* Subsect. *Obtusipetalum*** | **Sect. *Bifolia*** | **Sect. *Bifolia*** | **Sect. *Bifolia*** | **Sect. *Bifolia*** |
| *C. guttatum* | *C. guttatum* | *C. guttatum* | *C. guttatum* | *C. guttatum* | *C. guttatum* |
|  |  | *C. yatabeanum* | *C. yatabeanum* (as *C. guttatum* ssp. *yatabeanum*) | *C. yatabeanum* | *C. yatabeanum* |
|  | **Series *Flabellinervia*** | **Sect. *Flabellinervia*** | **Sect. *Flabellinervia*** | **Sect. *Flabellinervia*** | **Sect. *Flabellinervia*** |
| *C. japonicum* | *C. japonicum* | *C. japonicum* | *C. japonicum* | *C. japonicum* | *C. japonicum* |
|  |  | *C. formosanum* | *C. formosanum* | *C. formosanum* | *C. formosanum* |
| ***Arietinum* group** | **Series *Arcuinervia* Sect. *Criosanthes*** | **Sect. *Arietinum*** | **Sect. *Arietinum*** | **Sect. *Arietinum*** | **Sect. *Arietina*** |
| *C. arietinum* | *C. arietinum* | *C. arietinum* | *C. arietinum* | *C. arietinum* | *C. arietinum* |
|  |  | *C. plectrochilum* | *C. plectrochilum* | *C. plectrochilum* | *C. plectrochilum* |
|  | ***Series Arcuinervia* Sect. *Trigonopedilum*** | **Sect. *Trigonopedia*** | **Sect. *Trigonopedia*** | **Sect. *Trigonopedia*** | **Sect. *Trigonopedium*** |
|  | *C. margaritaceum* | *C. margaritaceum* | *C. margaritaceum* | *C. margaritaceum* | *C. margaritaceum* |
|  |  | *C. lichiangense* | *C. lichiangense* | *C. lichiangense* | *C. lichiangense* |
|  |  | *C. wumengense* |  | *C. wumengense* | *C. wumengense* |
|  | *C. fargesii* (as *C. ebracteatum*) | *C. fargesii* | *C. fargesii* (as *C. margaritaceum* ssp. *fargesii*) | *C. fargesii* | *C. fargesii* |
|  |  |  | *C. lentiginosum* (as *C. lichiangense* ssp. *lentiginosum*) | *C. lentiginosum* | *C. lentiginosum* |
|  |  |  | *C. sichuanense* (as *C. margaritaceum* ssp. *sichuanense*) | *C. sichuanense* | *C. sichuanense* |
|  |  |  |  |  | *C. daweishanense* |
|  |  |  |  |  | *C. malipoense* |
|  | **Series *Arcuinervia*  Sect. *Enantiopedilum*** |  | **Sect. *Sinopedilum*** | **Sect. *Sinopedilum*** | **Sect. *Sinopedilum*** |
|  | *C. micranthum* | *C. micranthum* | *C. micranthum* | *C. micranthum* | *C. micranthum* |
|  |  | *C. bardolphianum* | *C. bardolphianum* | *C. bardolphianum* | *C. bardolphianum* |
|  |  | *C. forrestii* | *C. forrestii* (as *C. bardolphianum* ssp. *forrestii*) | *C. forrestii* | *C. forrestii* |
|  |  |  |  |  |  |

Table S2: *Cypripedium* specimens sampled from the Botanical Collection at Oberhof, associated with the BGM, and the herbarium M.

| **Taxon** | **Accepted Name ^a^** | **Lab No. ^b^** | **Collection/ Herbarium** | **Collection Number** | **Collection Location** | **SRA Accession No.** |
| --- | --- | --- | --- | --- | --- | --- |
| *Cypripedium acaule* Aiton | *-* | 74* | Oberhof | - | - | SRR28779999 |
| *Cypripedium amesianum* Schltr. | *C. yunnanense* Franch. | 35* | Oberhof | - | - | SRR28779989 |
| *Cypripedium bardolphianum* W. W. Sm. & Farrer | *-* | 36 | Oberhof | 2023/1214-1 | - | SRR28779988 |
| *Cypripedium calceolus* L. | *-* | 1* | Oberhof | 2023/1209-1 | - | SRR28780041 |
| *Cypripedium calceolus* L. | *-* | 44* | Oberhof | 2023/1256-1 | - | SRR28780011 |
| *Cypripedium calcicola* Schltr. | *-* | 2* | Oberhof | 2023/1193-1 | - | SRR28780024 |
| *Cypripedium californicum* A. Gray | *-* | 3* | Oberhof | - | - | SRR28780037 |
| *Cypripedium candidum* Muehl. ex Willd. | *-* | 4* | Oberhof | - | - | SRR28780002 |
| *Cypripedium cordigerum* D. Don | *-* | 5* | Oberhof | 2023/1132-1 | - | SRR28780015 |
| *Cypripedium debile* Rchb.f. | - | 77* | Oberhof | 2023/1228-1 | - | SRR28779996 |
| *Cypripedium fargesii* Franch. | *-* | 47* | Oberhof | 2023/1218-1 | - | SRR28780032 |
| *Cypripedium farreri* W. W. Sm. | *-* | 6* | Oberhof | 2023/1213-1 | - | SRR28780028 |
| *Cypripedium fasciolatum* Franch. | *-* | 7* | Oberhof | 2023/1155-1 | - | SRR28779993 |
| *Cypripedium flavum* P. F. Hunt & Summerh. | *-* | 8* | Oberhof | 2023/1172-1 | - | SRR28779982 |
| *Cypripedium formosanum* Hayata | *-* | 9* | Oberhof | 2023/1202-1 | - | SRR28779981 |
| *Cypripedium franchetii* Wilson | *-* | 10* | Oberhof | 2023/1150-1 | - | SRR28779980 |
| *Cypripedium froschii* Perner | *-* | 11* | Oberhof | 2023/1126-1 | - | SRR28780023 |
| *Cypripedium guttatum* Swartz | *-* | 12* | Oberhof | 2023/1261-1 | - | SRR28780022 |
| *Cypripedium henryi* Rolfe | *-* | 13 | Oberhof | 2023/1179-1 | - | SRR28780021 |
| *Cypripedium himalaicum* Rolfe | *-* | 45* | Oberhof | - | - | SRR28780010 |
| *Cypripedium irapeanum* La Llave & Lex. | *-* | 14* | Oberhof | - | - | SRR28780020 |
| *Cypripedium japonicum* Thunb. | *-* | 15* | Oberhof | 2023/1147-1 | - | SRR28780019 |
| *Cypripedium kentuckiense* C. F. Reed | *-* | 16* | Oberhof | 2023/1166-1 | - | SRR28780018 |
| *Cypripedium lentiginosum* P. J. Cribb & S. C. Chen | *-* | 37 | Oberhof | - | - | SRR28779987 |
| *Cypripedium lichiangense* S. C. Chen & P. J. Cribb | *-* | 39 | Oberhof | 2023/ 1223-1 | - | SRR28779985 |
| *Cypripedium macranthos* Sw*.* var. *macranthos* | *-* | 17* | Oberhof | 2023/1242-1 | - | SRR28780017 |
| *Cypripedium macranthos* var. *alba* | Probably *C. macranthos* var. *albiflorum* Makino (now synonym of *C. macranthos* Sw*.* var. *macranthos*)*,* or *C. macranthos* var. *album* Mandl | 18* | Oberhof |  | - | SRR28780040 |
| *Cypripedium macranthos ‘*var. *hotei-atsmorianum* Sadovsky*’* | *-* | 19* | Oberhof | 2023/1176-1 | - | SRR28780039 |
| *Cypripedium macranthos* ‘var. *rebunense* (Kudo) Ohwi*’* | *-* | 20* | Oberhof | 2023/1151-1 | - | SRR28780038 |
| *Cypripedium macranthos* var. *speciosum* (Rolfe) Koidz. | *-* | 21* | Oberhof | 2023/1186-1 | - | SRR28780036 |
| *Cypripedium macranthos* ‘var. *taiwanianum* (Masam.) Maekwa’ | *-* | 46* | Oberhof | 2023/1219-1 | - | SRR28780009 |
| *Cypripedium micranthum* Franch. | *-* | 22* | Oberhof | 2023/1194-1 | - | SRR28780035 |
| *Cypripedium montanum* Douglas ex Lindl. | *-* | 23* | Oberhof | 2023/1212-1 | - | SRR28780034 |
| *Cypripedium parviflorum* Salisb var. *parviflorum* | *-* | 75* | Oberhof | 2023/1216-1 | - | SRR28779998 |
| *Cypripedium parviflorum* Salisb var. *makasin* (Farw.) C. J. Sheviak | *C. parviflorum* Salisb var. *parviflorum* | 24* | Oberhof | 2023/1192-1 | - | SRR28780033 |
| *Cypripedium parviflorum* Salisb*. var. pubescens* (Willd.) | *-* | 25* | Oberhof | 2023/1144-1 | - | SRR28780008 |
| *Cypripedium parviflorum* Salisb. var. *pubescens (*Willd.) O. W. Knigh*t forma planipetalum* (Fernald) P. J. Cribb | *-* | 26* | Oberhof | 2023/1210-1 | - | SRR28780007 |
| *Cypripedium passerinum* Richardson | *-* | 42* | Oberhof | 2023/1269-1 | - | SRR28780013 |
| *Cypripedium plectrochilum* Franch. | *-* | 27* | Oberhof | 2023/1217-1 | - | SRR28780006 |
| *Cypripedium reginae* Walter | *-* | 40* | Oberhof | 2023/1196-1 | - | SRR28780016 |
| *Cypripedium reginae* var. *alba* | *C. reginae* var. *album* (Aiton) Rolfe | 41* | Oberhof | 2023/1162-1 | - | SRR28780014 |
| *Cypripedium segawai* Masam. | *-* | 28* | Oberhof | 2023/1170-1 | - | SRR28780005 |
| *Cypripedium shanxiense* S. C. Chen | *-* | 29* | Oberhof | - | - | SRR28780004 |
| *Cypripedium subtropicum* S. C. Chen & K. Y. Lang | *-* | 76* | Oberhof | 2023/1233-1 | - | SRR28779997 |
| *Cypripedium tibeticum* King ex Rolfe | *-* | 30* | Oberhof | 2023/1127-1 | - | SRR28780003 |
| *Cypripedium wardii* Rolfe | *-* | 38* | Oberhof | - | - | SRR28779986 |
| *Cypripedium yatabeanum* Makino | *-* | 31* | Oberhof | 2023/1260-1 | - | SRR28780001 |
| *Cypripedium yunnanense* Franch. | *-* | 32* | Oberhof | - | - | SRR28779992 |
| *Cypripedium* × *alaskanum* P. M. Br. | *-* | 43* | Oberhof | 2023/1265-1 | - | SRR28780012 |
| *Cypripedium* × *columbianum* Sheviak | *-* | 33* | Oberhof | 2023/1130-1 | - | SRR28779991 |
| *Cypripedium* × *ventricosum* Sw. | *-* | 34* | Oberhof | 2023/1133-1 | - | SRR28779990 |
| *Cypripedium acaule* Aiton | *-* | 48* | M | Eric A. Bourdo, Jr. 28,450 (1974) | Michigan, U.S.A. | SRR28780031 |
| *Cypripedium californicum* A. Gray | *-* | 57* | M | Mary F. Spencer s.n. (1916) | California, U.S.A. | SRR28780027 |
| *Cypripedium calceolus* L. var*. parviflorum* (Salsib) Fernald | *C. parviflorum* Salisb var. *parviflorum* | 53* | M | W. J. Dress 5965 (1958) | New York, U.S.A. | SRR28780029 |
| *Cypripedium calceolus* var. *pubescens* (Willd.) Correll | *Cypripedium parviflorum* Salisb. *var. pubescens* (Willd.) | 69* | M | Eric A. Bourdo 32295 (1976) | Michigan, U.S.A. | SRR28780000 |
| *Cypripedium debile* Rchb.f. | - | 62 | M | B. Dickoré 14259 (1996) | NW Yunnan, China | SRR28780026 |
| *Cypripedium himalaicum* Rolfe | - | 65* | M | F. Lobbichler 121 (1955) | Manangbhot, Nepal | SRR28780025 |
| *Cypripedium passerinum* Richardson var. *passerinum* | **-** | 52 | M | W. J. Cody 4044 (1950) | Mackenzie District, Northwest Territories, Canada | SRR28780030 |

^a^ According to Frosch and Cribb (2012).
^b^ Lab numbers followed by an asterisk (“*”) indicate samples included in the cpDNA tree.

Table S3: *Cypripedium* DNA samples provided by the DNA collection of the Kew Royal Botanic Gardens.

| **Taxon** | **Lab No. ^a^** | **Collector(s)** | **Collector Number** | **Collection Date** | **Country** | **Voucher** | **MWC** | **Dnald** | **SRA Accession No.** |
| --- | --- | --- | --- | --- | --- | --- | --- | --- | --- |
| *Cypripedium bardolphianum* W. W. Sm. & Farrer | 83* | Huang Long Si | s. n. | 1997-06 | China | Huang Long Si | 5722 | 5722 | SRR28779995 |
| *Cypripedium fasciculatum* Kellogg ex S. Watson | 88* | H. Peruv | (4/6/1996) | - | USA, Washington State, Killitas County, Mineral Springs. | H. Peruv (4/6/1996) | O-1269 | 5269 | SRR28779983 |
| *Cypripedium fasciolatum* Franch. | 84* | Huang Long Si | s. n. | 1997-06 | China | Huang Long Si | 5723 | 5723 | SRR28779994 |
| *Cypripedium lichiangense S. C. Chen & P. J. Cribb* | 87* | P. Cribb | (6/8/92) | - | Unknown | P. Cribb (6/8/92) | O-953 | 4953 | SRR28779984 |

^a^ Lab numbers followed by an asterisk (“*”) indicate samples included in the cpDNA tree.

Table S4: List publicly available orchid sequence data used in this study.

| **Taxon** | **Accepted Name** ^a^ | **Source/Bioproject  Accession Number** | **NCBI Accession  Number** ^b^ | **Sequence type** ^c^ |
| --- | --- | --- | --- | --- |
| *Apostasia shenzhenica* Z. J. Liu & L. J. Chen | - | Zhang *et al.* (2017) | - | genome* |
| *Apostasia shenzhenica* Z. J. Liu & L. J. Chen | - | PRJNA927338 | NC039812 | chloroplast genome |
| *Cypripedium acaule* Aiton | - | PRJNA412930 | SRX3240244 | transcriptome*✝ |
| *Cypripedium bardolphianum* W. W. Sm. & Farrer | - | PRJNA479379 | SRX4336453 | transcriptome*✝ |
| *Cypripedium bardolphianum* W. W. Sm. & Farrer | - | Hu *et al.,* 2022 | OL741711 | chloroplast genome |
| *Cypripedium calceolus* L. | - | PRJNA927338 | NC045400 | chloroplast genome |
| *Cypripedium debile* Rchb.f. | - | PRJNA838021 | SRX15379764 | genome skimming |
| *Cypripedium debile* Rchb.f. | - | PRJNA927338 | NC063681 | chloroplast genome |
| *Cypripedium fargesii* Franch. | - | PRJNA479379 | SRX4336442 | transcriptome*✝ |
| *Cypripedium fargesii* Franch. | - | PRJNA927338 | NC084418 | chloroplast genome |
| *Cypripedium farreri* W. W. Sm. | - | Hu *et al.,* 2022 | OM066273 | chloroplast genome |
| *Cypripedium fasciolatum* Franch. | - | Hu *et al.,* 2022 | OM066274 | chloroplast genome |
| *Cypripedium flavum* P. F. Hunt & Summerh. | - | PRJNA479379 | SRX4336448 | transcriptome*✝ |
| *Cypripedium flavum* P. F. Hunt & Summerh. | - | Hu *et al.,* 2022 | OM066275 | chloroplast genome |
| *Cypripedium formosanum* Hayata | - | PRJNA277578 | SRX911751 | transcriptome*✝ |
| *Cypripedium formosanum* Hayata | - | PRJNA927338 | NC026772 | chloroplast genome |
| *Cypripedium forrestii* P. J. Cribb | - | PRJNA1029356 | SRX22160934 | transcriptome✝ |
| *Cypripedium guttatum* Swartz | - | Hu *et al.,* 2022 | OM066278 | chloroplast genome |
| *Cypripedium henryi* Rolfe | - | Hu *et al.,* 2022 | OM066279 | chloroplast genome |
| *Cypripedium japonicum* Thunb. | - | Hu *et al.,* 2022 | OM066280 | chloroplast genome |
| *Cypripedium macranthos* ‘var. *rebunense* (Kudo) Ohwi*’* | - | PRJDB15443 | DRX436158 | transcriptome |
| *Cypripedium margaritaceum* Franch. | - | PRJNA479379 | SRX4336454 | transcriptome*✝ |
| *Cypripedium micranthum* Franch. | - | PRJNA479379 | SRX4336451 | transcriptome*✝ |
| *Cypripedium lentiginosum* P. J. Cribb & S. C. Chen | - | PRJNA479379 | SRX4336441 | transcriptome*✝ |
| *Cypripedium lichiangense* S. C. Chen & P. J. Cribb | - | PRJNA1029356 | SRX22160950 | transcriptome✝ |
| *Cypripedium lichiangense* S. C. Chen & P. J. Cribb | - | PRJNA927338 | NC084419 | chloroplast genome |
| *Cypripedium palangshanense* T. Tang & F. T. Wang | - | PRJNA838021 | SRX15379763 | genome skimming |
| *Cypripedium palangshanense* T. Tang & F. T. Wang | - | PRJNA927338 | NC063680 | chloroplast genome |
| *Cypripedium plectrochilum Franch.* | - | Hu *et al.,* 2022 | OM066284 | chloroplast genome |
| *Cypripedium sichuanense* Perner | - | PRJNA479379 | SRX4336445 | transcriptome*✝ |
| *Cypripedium sichuanense* Perner | - | PRJNA927338 | NC084420 | chloroplast genome |
| *Cypripedium subtropicum* S. C. Chen & K. Y. Lang | - | PRJNA927338 | NC053551 | chloroplast genome |
| *Cypripedium singchii* Z. J. Liu & L. J. Chen | *Cypripedium subtropicum* S. C.  Chen & K. Y. Lang | PRJNA479379 | SRX4336446 | transcriptome*✝ |
| *Cypripedium × ventricosum Sw.* | - | Hu *et al.,* 2022 | OM066286 | chloroplast genome |
| *Dendrobium catenatum* Lindl. | *Dendrobium officinale* Kimura  & Migo | Zhang *et al.* (2016) | - | genome* |
| *Dendrobium catenatum* Lindl. | *Dendrobium officinale* Kimura  & Migo | PRJNA927338 | NC037361 | chloroplast genome |
| *Mexipedium xerophyticum* (Soto Arenas, Salazar & Hágsater) V. A. Albert & M. W. Chase | - | PRJNA412930 | SRX3240239 | transcriptome* |
| *Mexipedium xerophyticum* (Soto Arenas, Salazar & Hágsater) V. A. Albert & M. W. Chase |  | PRJNA927338 | NC069868 | chloroplast genome |
| *Paphiopedilum callosum* Pfitzer | - | PRJNA412930 | SRX3240238 | transcriptome* |
| *Paphiopedilum callosum* Pfitzer | - | PRJNA927338 | NC069960 | chloroplast genome |
| *Paphiopedilum concolor* Pfitzer | - | PRJNA252662 | SRX601820 | transcriptome* |
| *Paphiopedilum concolor* Pfitzer | - | PRJNA927338 | NC069964 | chloroplast genome |
| *Paphiopedilum hirsutissimum* Pfitzer | - | PRJNA252662 | SRX601821 | transcriptome* |
| *Paphiopedilum hirsutissimum* Pfitzer | - | PRJNA927338 | NC050871 | chloroplast genome |
| *Phalaenopsis equestris* (Schauer) Rchb.f. | - | Cai *et al.* (2015) | - | genome* |
| *Phalaenopsis equestris* (Schauer) Rchb.f. | - | PRJNA927338 | NC017609 | chloroplast genome |
| *Phragmipedium lindleyanum* (R. H. Schomb. ex Lindl.) Rolfe | - | PRJNA412930 | SRX3240237 | transcriptome*✝ |
| *Selenipedium aequinoctiale* Garay | - | PRJNA412930 | SRX3240236 | transcriptome*✝ |
| *Vanilla planifolia* Andrews | - | Piet *et al.* (2022) | - | genome* |
| *Vanilla planifolia* Andrews | - | PRJNA927338 | NC026778 | chloroplast genome |
| *Vanilla shenzhenica* Z. J. Liu & S. C. Chen | - | PRJNA310678 | SRX2938656 | transcriptome*✝ |

^a^According to Frosch and Cribb (2012) for *Cypripedium*, or POWO (2023) for the rest.

^b^Accession numbers starting with “SR” and “DR” were acquired from the SRA database of NCBI and accession numbers starting with “NC”, “OM” and “OL” were acquired from NCBI GenBank.
^c^ Transcriptomes and genomes with an asterisk (“*”) were used to create the set of references to improve gene extraction from the target enrichment sequence data. Transcriptomes with a cross (“✝”) were included in the cpDNA tree.

Table S5: Modifications to the Macherey-Nagel NucleoSpin Plant II kit: Genomic DNA from plant (Macherey-Nagel – 07/2014, Rev.09) protocol.

| **Step Nr.** | **Modified instructions** |
| --- | --- |
| **1** | Approximately 25 mg (or less, in cases where <25 mg was available) of silica-dried or herbarium leaf material was fragmented with forceps and placed into a 2 mL microcentrifuge tube with two glass beads (6 mm in diameter). Next, the samples were homogenized using a Retsch Tissuelyser for 5 –10 mins at 30/s, until the tissue turned into very fine powder. |
| **2a** | The tubes containing the homogenized tissue were centrifuged shortly. 600 μL Buffer PL1 and 10 μL RNase A (from the MN kit) were added, and the samples were vortexed thoroughly. The suspensions were incubated on a thermomixer for 60 mins at 65 °C and 400 rpm, and they were shortly vortexed every 15 mins. |
| **6** | **1^st^ wash**: 400 μL Buffer PW1 were added to the columns, and they were centrifuged for 1 min at 11,000 x g. The flow-through was discarded.  **2^nd^ wash**: 600 μL Buffer PW2 were added to the columns, and they were centrifuged for 1 min at 11,000 x g. The flow-through was discarded.  **3^rd^ wash**: 350 μL Buffer PW2 were added to the columns, and they were centrifuged for 1 min at 11,000 x g. The flow-through was discarded.  **4^th^ wash**: 200 μL Buffer PW2 were added to the columns, and they were centrifuged for 2 min at 11,000 x g. The flow-through was discarded. |
| **7** | **Eluate A:** The column was placed in a new 1.5 mL microcentrifuge tube, and 50 μL Buffer PE (65 °C) was pipetted onto the membrane. The samples were incubated on a thermomixer for 5 mins at 65 °C and 300 rpm. Then, they were centrifuged for 1 min at 11,000 x g to elute the DNA.  **Eluate B:** The previous step was repeated by placing the column into another 1.5 mL microcentrifuge tube to produce a second eluate with another 50 μL Buffer PE (65 °C), to avoid decreasing the concentration of the first eluate.  The eluates were stored at -20 °C until further use. |

Table S6: *Cypripedium* specimens sampled from the Botanical Collection at Oberhof, associated with the BGM, for the production of transcriptomic data to be subsequently used in the Ks plots analysis.

| **Taxon** | **Lab No.** | **Collection Number** | **SRA Accession No.** |
| --- | --- | --- | --- |
| *Cypripedium californicum* A. Gray | 95 | 2023/1173-1 | SRR28803710 |
| *Cypripedium guttatum* Swartz | 12 | 2023/1261-1 | SRR28803716 |
| *Cypripedium henryi* Rolfe | 97 | 2023/1158-1 | SRR28803708 |
| *Cypripedium irapeanum* La Llave & Lex. | 94 | - | SRR28803711 |
| *Cypripedium kentuckiense* C. F. Reed | 98 | 2023/1237-1 | SRR28803707 |
| *Cypripedium parviflorum* Salisb var. *parviflorum* | 90 | 2023/1249-1 | SRR28803714 |
| *Cypripedium parviflorum* Salisb*. var. pubescens* (Willd.) | 91 | 2023/1138-1 | SRR28803713 |
| *Cypripedium plectrochilum* Franch. | 92 | 2023/1278-1 | SRR28803712 |
| *Cypripedium segawai* Masam. | 96 | 2023/1230-1 | SRR28803709 |
| *Cypripedium yatabeanum* Makino | 31 | 2023/1260-1 | SRR28803715 |

Table S7: Final occupancy statistics for 913 orthologous nuclear loci and characters (bp) per specimen following the concatenation step.

| **Taxon ID** ^a^ | **#of orthologs** | **# of characters** | **% of orthologs** | **% of characters** |
| --- | --- | --- | --- | --- |
| C_palangshanense_gks | 18 | 12030 | 0.02 | 0.01 |
| C_debile_62 | 53 | 35231 | 0.06 | 0.04 |
| C_debile_gks | 60 | 20319 | 0.07 | 0.02 |
| C_passerinum_var_passerinum_52 | 181 | 128217 | 0.20 | 0.13 |
| C_himalaicum_65 | 236 | 184170 | 0.26 | 0.19 |
| C_calceolus_var_par_53 | 283 | 209943 | 0.31 | 0.21 |
| C_calceolus_var_pub_69 | 310 | 230140 | 0.34 | 0.24 |
| C_calceolus_44 | 441 | 433171 | 0.48 | 0.44 |
| C_acaule_48 | 442 | 354653 | 0.48 | 0.36 |
| C_lichiangense_39 | 449 | 391080 | 0.49 | 0.40 |
| C_henryi_13 | 455 | 405944 | 0.50 | 0.41 |
| C_lentiginosum_37 | 470 | 422874 | 0.51 | 0.43 |
| C_franchetii_10 | 493 | 457599 | 0.54 | 0.47 |
| C_froschii_11 | 508 | 469179 | 0.56 | 0.48 |
| C_shanxiense_29 | 535 | 543326 | 0.59 | 0.56 |
| C_californicum_57 | 545 | 432531 | 0.60 | 0.44 |
| C_bardolphianum_36 | 545 | 508112 | 0.60 | 0.52 |
| C_formosanum_09 | 561 | 532424 | 0.61 | 0.54 |
| C_guttatum_12 | 572 | 514172 | 0.63 | 0.53 |
| C_segawai_28 | 609 | 585758 | 0.67 | 0.60 |
| C_lichiangense_87 | 610 | 563905 | 0.67 | 0.58 |
| C_montanum_23 | 620 | 654321 | 0.68 | 0.67 |
| C_amesianum_35 | 626 | 596568 | 0.69 | 0.61 |
| C_acaule_74 | 627 | 568915 | 0.69 | 0.58 |
| C_par_var_pub_f_planipetalum_26 | 634 | 646728 | 0.69 | 0.66 |
| C_x_columbianum_33 | 643 | 643758 | 0.70 | 0.66 |
| C_cordigerum_05 | 646 | 670949 | 0.71 | 0.69 |
| C_kentuckiense_16 | 646 | 654250 | 0.71 | 0.67 |
| C_par_var_makasin_24 | 646 | 659109 | 0.71 | 0.67 |
| C_par_var_parviflorum_75 | 648 | 660305 | 0.71 | 0.67 |
| C_par_var_pub_25 | 649 | 654967 | 0.71 | 0.67 |
| C_passerinum_42 | 651 | 632800 | 0.71 | 0.65 |
| C_candidum_04 | 661 | 676019 | 0.72 | 0.69 |
| C_mac_var_hotei-atsumorianum_19 | 670 | 683432 | 0.73 | 0.70 |
| C_tibeticum_30 | 671 | 659175 | 0.73 | 0.67 |
| C_farreri_06 | 671 | 670480 | 0.73 | 0.69 |
| C_x_ventricosum_34 | 671 | 651993 | 0.73 | 0.67 |
| C_yunnanense_32 | 674 | 671975 | 0.74 | 0.69 |
| C_himalaicum_45 | 676 | 683549 | 0.74 | 0.70 |
| C_fargesii_47 | 678 | 684155 | 0.74 | 0.70 |
| C_micranthum_22 | 679 | 675347 | 0.74 | 0.69 |
| C_fasciolatum_07 | 680 | 669205 | 0.74 | 0.68 |
| C_fasciolatum_84 | 681 | 688907 | 0.75 | 0.70 |
| C_calceolus_01 | 682 | 657206 | 0.75 | 0.67 |
| C_mac_var_speciosum_21 | 684 | 684491 | 0.75 | 0.70 |
| C_mac_var_alba_18 | 685 | 691253 | 0.75 | 0.71 |
| C_mac_var_taiwanianum_46 | 687 | 686315 | 0.75 | 0.70 |
| C_reginae_var_alba_41 | 689 | 676238 | 0.75 | 0.69 |
| C_mac_var_rebunense_20 | 693 | 703280 | 0.76 | 0.72 |
| C_subtropicum_76 | 693 | 688927 | 0.76 | 0.70 |
| C_wardii_38 | 695 | 684415 | 0.76 | 0.70 |
| C_calcicola_02 | 697 | 701028 | 0.76 | 0.72 |
| C_mac_var_mac_17 | 698 | 692451 | 0.76 | 0.71 |
| C_bardolphianum_83 | 700 | 696571 | 0.77 | 0.71 |
| C_reginae_40 | 705 | 693617 | 0.77 | 0.71 |
| C_debile_77 | 707 | 670775 | 0.77 | 0.69 |
| C_flavum_08 | 708 | 697712 | 0.78 | 0.71 |
| C_fasciculatum_88 | 709 | 701729 | 0.78 | 0.72 |
| C_yattabeanum_31 | 712 | 708863 | 0.78 | 0.72 |
| C_mac_var_rebunense_trp | 718 | 786843 | 0.79 | 0.80 |
| C_japonicum_15 | 724 | 724032 | 0.79 | 0.74 |
| C_x_alaskanum_43 | 733 | 719207 | 0.80 | 0.73 |
| C_californicum_03 | 739 | 751130 | 0.81 | 0.77 |
| C_singchii_trp | 743 | 755455 | 0.81 | 0.77 |
| C_lentiginosum_trp | 748 | 750604 | 0.82 | 0.77 |
| C_sichuanense_trp | 757 | 771902 | 0.83 | 0.79 |
| C_bardolphianum_trp | 760 | 819755 | 0.83 | 0.84 |
| C_plectrochilon_27 | 760 | 769505 | 0.83 | 0.79 |
| C_forrestii_trp | 764 | 819863 | 0.84 | 0.84 |
| C_micranthum_trp | 765 | 811088 | 0.84 | 0.83 |
| C_fargesii_trp | 770 | 830654 | 0.84 | 0.85 |
| C_irapeanum_14 | 770 | 803092 | 0.84 | 0.82 |
| C_margaritaceum_trp | 773 | 839348 | 0.85 | 0.86 |
| C_lichiangense_trp | 777 | 845618 | 0.85 | 0.86 |
| C_formosanum_trp | 782 | 836789 | 0.86 | 0.85 |
| C_flavum_trp | 784 | 845858 | 0.86 | 0.86 |
| C_acaule_trp | 795 | 837286 | 0.87 | 0.86 |
| Paphiopedilum_callosum_trp | 820 | 827313 | 0.90 | 0.85 |
| Apostasia_shenzhenica_gen | 838 | 819269 | 0.92 | 0.84 |
| Vanilla_planifolia_gen | 840 | 826307 | 0.92 | 0.84 |
| Selenipedium_aequinoctiale_trp | 851 | 883803 | 0.93 | 0.90 |
| Paphiopedilum_hirsutissimum_trp | 853 | 891870 | 0.93 | 0.91 |
| Paphiopedilum_concolor_trp | 862 | 901932 | 0.94 | 0.92 |
| Mexipedium_xerophyticum_trp | 871 | 916935 | 0.95 | 0.94 |
| Phragmipedium_lindleyanum_trp | 875 | 925251 | 0.96 | 0.95 |
| Vanilla_shenzhenica_trp | 875 | 849137 | 0.96 | 0.87 |
| Phalaenopsis_equestris_gen | 898 | 928175 | 0.98 | 0.95 |
| Dendrobium_catenatum_gen | 900 | 922223 | 0.99 | 0.94 |

^a^ Taxon ID is comprised of (a) the taxon name followed by the assigned Lab No. for samples collected from herbarium M, the Botanical Collection at Oberhof associated with the BGM or provided by the Kew Royal Botanical Gardens, or (b) the taxon name followed by the sequence type (trp = transcriptome, gen = genome, gks = genome skimming) for publicly available orchid sequences. Samples whose taxon ID starts with “C_” belong to the genus *Cypripedium.* See Supplementary Data Tables S2, S3, and S4 for more details.

Table S8: Final occupancy statistics of the 80 orthologous chloroplast loci and characters (bp) per specimen following the concatenation step.

| **Taxon ID** ^a^ | **# of orthologs** | **# of characters** | **% of orthologs** | **% of characters** |
| --- | --- | --- | --- | --- |
| Cypripedium_himalaicum_65 | 39 | 32205 | 0.49 | 0.47 |
| Cypripedium_parviflorum_var_parviflorum_75 | 43 | 44406 | 0.54 | 0.65 |
| Cypripedium_froschii_11 | 45 | 46698 | 0.56 | 0.68 |
| Cypripedium_formosanum_09 | 47 | 46758 | 0.59 | 0.68 |
| Cypripedium_acaule_48 | 56 | 45900 | 0.70 | 0.67 |
| Cypripedium_franchetii_10 | 57 | 52008 | 0.71 | 0.76 |
| Cypripedium_macranthos_var_taiwanianum_46 | 58 | 52407 | 0.73 | 0.76 |
| Cypripedium_debile_77 | 58 | 51288 | 0.73 | 0.75 |
| Cypripedium_calceolus_var_pubescens_69 | 60 | 50196 | 0.75 | 0.73 |
| Cypripedium_wardii_38 | 62 | 51981 | 0.78 | 0.76 |
| Cypripedium_x_ventricosum_34 | 62 | 54819 | 0.78 | 0.80 |
| Cypripedium_acaule_74 | 62 | 55635 | 0.78 | 0.81 |
| Cypripedium_himalaicum_45 | 63 | 56079 | 0.79 | 0.81 |
| Cypripedium_calceolus_44 | 63 | 55872 | 0.79 | 0.81 |
| Vanilla_shenzhenica_trp | 63 | 33906 | 0.79 | 0.49 |
| Cypripedium_parviflorum_var_makasin_24 | 64 | 57894 | 0.80 | 0.84 |
| Cypripedium_x_columbianum_33 | 64 | 54978 | 0.80 | 0.80 |
| Cypripedium_japonicum_15 | 65 | 56043 | 0.81 | 0.81 |
| Cypripedium_guttatum_12 | 65 | 58968 | 0.81 | 0.86 |
| Cypripedium_singchii_trp | 66 | 44634 | 0.83 | 0.65 |
| Cypripedium_segawai_28 | 66 | 57753 | 0.83 | 0.84 |
| Cypripedium_macranthos_var_rebunense_20 | 67 | 59925 | 0.84 | 0.87 |
| Cypripedium_parviflorum_var_pubescens_25 | 67 | 59844 | 0.84 | 0.87 |
| Vanilla_planifolia_NC026778 | 67 | 55149 | 0.84 | 0.80 |
| Cypripedium_reginae_var_alba_41 | 68 | 58578 | 0.85 | 0.85 |
| Cypripedium_macranthos_var_alba_18 | 68 | 60402 | 0.85 | 0.88 |
| Cypripedium_flavum_08 | 68 | 58746 | 0.85 | 0.85 |
| Cypripedium_irapeanum_14 | 68 | 57177 | 0.85 | 0.83 |
| Paphiopedilum_hirsutissimum_NC050871 | 70 | 60186 | 0.88 | 0.87 |
| Paphiopedilum_callosum_NC069960 | 70 | 59376 | 0.88 | 0.86 |
| Cypripedium_tibeticum_30 | 70 | 60051 | 0.88 | 0.87 |
| Mexipedium_xerophyticum_NC069868 | 70 | 59997 | 0.88 | 0.87 |
| Cypripedium_yunnanense_32 | 71 | 61008 | 0.89 | 0.89 |
| Cypripedium_parviflorum_var_pubescens_f_planipetalum_26 | 72 | 63021 | 0.90 | 0.92 |
| Cypripedium_cordigerum_05 | 72 | 63546 | 0.90 | 0.92 |
| Cypripedium_kentuckiense_16 | 72 | 62937 | 0.90 | 0.91 |
| Cypripedium_shanxiense_29 | 72 | 60678 | 0.90 | 0.88 |
| Phragmipedium_lindleyanum_trp | 73 | 59802 | 0.91 | 0.87 |
| Cypripedium_sichuanense_trp | 73 | 48675 | 0.91 | 0.71 |
| Phalaenopsis_equestris_NC017609 | 73 | 60312 | 0.91 | 0.88 |
| Cypripedium_macranthos_var_hotei-atsumorianum_19 | 73 | 63402 | 0.91 | 0.92 |
| Cypripedium_micranthum_22 | 74 | 66276 | 0.93 | 0.96 |
| Cypripedium_debile_NC063681 | 74 | 62445 | 0.93 | 0.91 |
| Cypripedium_plectrochilon_27 | 74 | 64959 | 0.93 | 0.94 |
| Cypripedium_fasciolatum_07 | 74 | 61299 | 0.93 | 0.89 |
| Cypripedium_fargesii_trp | 74 | 49977 | 0.93 | 0.73 |
| Cypripedium_macranthos_var_speciosum_21 | 74 | 63093 | 0.93 | 0.92 |
| Paphiopedilum_concolor_NC069964 | 74 | 61983 | 0.93 | 0.90 |
| Cypripedium_reginae_40 | 74 | 62031 | 0.93 | 0.90 |
| Cypripedium_palangshanense_NC063680 | 75 | 62778 | 0.94 | 0.91 |
| Cypripedium_amesianum_35 | 75 | 61542 | 0.94 | 0.89 |
| Cypripedium_calcicola_02 | 75 | 62193 | 0.94 | 0.90 |
| Cypripedium_farreri_06 | 75 | 65538 | 0.94 | 0.95 |
| Cypripedium_forrestii_trp | 75 | 47823 | 0.94 | 0.69 |
| Cypripedium_micranthum_trp | 76 | 52704 | 0.95 | 0.77 |
| Cypripedium_calceolus_var_parviflorum_53 | 76 | 60942 | 0.95 | 0.89 |
| Cypripedium_candidum_04 | 76 | 64689 | 0.95 | 0.94 |
| Cypripedium_lentiginosum_trp | 76 | 51645 | 0.95 | 0.75 |
| Apostasia_shenzhenica_NC039812 | 76 | 63060 | 0.95 | 0.92 |
| Cypripedium_margaritaceum_trp | 76 | 57273 | 0.95 | 0.83 |
| Dendrobium_catenatum_NC037361 | 77 | 62532 | 0.96 | 0.91 |
| Cypripedium_bardolphianum_trp | 77 | 56571 | 0.96 | 0.82 |
| Cypripedium_lichiangense_trp | 77 | 56112 | 0.96 | 0.82 |
| Cypripedium_flavum_trp | 77 | 54714 | 0.96 | 0.79 |
| Cypripedium_formosanum_trp | 77 | 51408 | 0.96 | 0.75 |
| Selenipedium_aequinoctiale_trp | 77 | 58287 | 0.96 | 0.85 |
| Cypripedium_montanum_23 | 77 | 66024 | 0.96 | 0.96 |
| Cypripedium_passerinum_42 | 78 | 64896 | 0.98 | 0.94 |
| Cypripedium_subtropicum_76 | 78 | 64956 | 0.98 | 0.94 |
| Cypripedium_macranthos_var_macranthos_17 | 78 | 65361 | 0.98 | 0.95 |
| Cypripedium_californicum_57 | 79 | 66963 | 0.99 | 0.97 |
| Cypripedium_flavum_OM066275 | 79 | 65835 | 0.99 | 0.96 |
| Cypripedium_bardolphianum_83 | 79 | 67968 | 0.99 | 0.99 |
| Cypripedium_x_ventricosum_OM066286 | 79 | 67641 | 0.99 | 0.98 |
| Cypripedium_formosanum_NC026772 | 79 | 65724 | 0.99 | 0.95 |
| Cypripedium_plectrochilum_OM066284 | 80 | 67509 | 1 | 0.98 |
| Cypripedium_fasciolatum_OM066274 | 80 | 67914 | 1 | 0.99 |
| Cypripedium_fargesii_47 | 80 | 66669 | 1 | 0.97 |
| Cypripedium_lichiangense_NC084419 | 80 | 67374 | 1 | 0.98 |
| Cypripedium_sichuanense_NC084420 | 80 | 66606 | 1 | 0.97 |
| Cypripedium_subtropicum_NC053551 | 80 | 67545 | 1 | 0.98 |
| Cypripedium_lichiangense_87 | 80 | 68130 | 1 | 0.99 |
| Cypripedium_bardolphianum_OL741711 | 80 | 67710 | 1 | 0.98 |
| Cypripedium_x_alaskanum_43 | 80 | 66792 | 1 | 0.97 |
| Cypripedium_yattabeanum_31 | 80 | 67653 | 1 | 0.98 |
| Cypripedium_japonicum_OM066280 | 80 | 67401 | 1 | 0.98 |
| Cypripedium_farreri_OM066273 | 80 | 68025 | 1 | 0.99 |
| Cypripedium_calceolus_NC045400 | 80 | 67764 | 1 | 0.98 |
| Cypripedium_fasciolatum_84 | 80 | 68289 | 1 | 0.99 |
| Cypripedium_fasciculatum_88 | 80 | 67986 | 1 | 0.99 |
| Cypripedium_californicum_03 | 80 | 67698 | 1 | 0.98 |
| Cypripedium_acaule_trp | 80 | 64854 | 1 | 0.94 |
| Cypripedium_fargesii_NC084418 | 80 | 65424 | 1 | 0.95 |
| Cypripedium_henryi_OM066279 | 80 | 67755 | 1 | 0.98 |
| Cypripedium_calceolus_01 | 80 | 65427 | 1 | 0.95 |
| Cypripedium_guttatum_OM066278 | 80 | 67626 | 1 | 0.98 |

^a^ Taxon ID is comprised of (a) the taxon name followed by the assigned Lab No. for samples collected from herbarium M, the Botanical Collection at Oberhof associated with the BGM or provided by the Kew Royal Botanical Gardens, (b) the taxon name followed by “trp” for publicly available orchid transcriptomes, or (c) the taxon name followed by the NCBI accession number for publicly available complete or partial chloroplast genome sequences for publicly available orchid sequences (see Supplementary Data Tables S2, S3, and S4 for more details).

Table S9: The α(𝑥) calculations of the anomaly zone test with the corresponding branch numbers and their branch lengths.

| **Branch 𝑥** | | **Branch 𝑦** | | **α(𝑥)** |
| --- | --- | --- | --- | --- |
| **Nr** | **length** | **Nr** | **length** |  |
| 17 | 4.598486 | 1 | 2.351982 | -0.402926145 |
| 1 | 2.351982 | 2 | 1.134936 | -0.3796615812450 |
| 2 | 1.134936 | 3 | 0.476353 | -0.3008610478330 |
| 3 | 0.476353 | 4 | 0.164628 | -0.1397532132990 |
| 4 | 0.164628 | 5 | 0.050474 | 0.1408893084410* |
| 5 | 0.050474 | 6 | 0.042781 | 0.6657161410210* |
| 6 | 0.042781 | 7 | 0.0094 | 0.7636745682490* |
| 7 | 0.0094 | 8 | 0.050667 | 1.9122368256400* |
| 8 | 0.050667 | 9 | 2.636816 | 0.6635291712650 |
| 9 | 2.636816 | 10 | 0.22772 | -0.3864359328600 |
| 9 | 2.636816 | 11 | 1.62219 | -0.3864359328600 |
| 12 | 0.184896 | 13 | 0.092595 | 0.1037583598950* |
| 13 | 0.092595 | 14 | 0.074992 | 0.3597101466500* |
| 14 | 0.074992 | 15 | 0.450694 | 0.4566492216810* |
| 6 | 0.042781 | 16 | 2.703795 | 0.76367456824900 |
| 18 | 0.014692 | 19 | 0.271572 | 1.5342160956800* |
| 20 | 0.178749 | 21 | 0.090561 | 0.1143601218400* |
| 21 | 0.090561 | 22 | 0.17481 | 0.3694667607310* |
| 21 | 0.090561 | 23 | 0.112374 | 0.3694667607310* |
| 22 | 0.17481 | 24 | 0.014068 | 0.1214406118860* |
| 24 | 0.014068 | 25 | 0.143974 | 1.5697848549700* |
| 24 | 0.014068 | 26 | 0.016977 | 1.5697848549700* |
| 27 | 0.066726 | 28 | 0.210933 | 0.5145939766250* |
| 29 | 0.101751 | 30 | 0.265228 | 0.3194486719450* |
| 31 | 0.150217 | 34 | 0.09857 | 0.1716905028390* |
| 32 | 0.139148 | 33 | 0.106984 | 0.1985105684870* |
| 34 | 0.09857 | 36 | 0.062525 | 0.3328029445320* |
| 34 | 0.09857 | 35 | 0.031944 | 0.3328029445320* |
| 35 | 0.031944 | 37 | 0.212367 | 0.9516747586560* |
| 37 | 0.212367 | 38 | 0.03756 | 0.0620216394774* |
| 38 | 0.03756 | 39 | 0.247431 | 0.8451225357780* |
| 39 | 0.247431 | 40 | 0.099602 | 0.0188788493410 |
| 40 | 0.099602 | 41 | 0.107745 | 0.3284008018810* |

“*” marks the α(𝑥) of all internode pairs that were found to be in the anomaly zone [i.e., 𝑦 < α(𝑥)]. For branch numbering, refer to Supplementary Data Figure S5.

| **Topology^a^** | **lnL** | **Number of tips** | **Parameters^b^** | **Loci** | **Number of hybridizations** | **Information criteria** | | | | | |
| --- | --- | --- | --- | --- | --- | --- | --- | --- | --- | --- | --- |
|  |  |  |  |  |  | **AIC** | **AICc** | **BIC** | **deltaAIC** | **deltaAICc** | **deltaBIC** |
| Network 1 | -556.90194 | 4 | 7 | 535 | 1 | 1127.80387 | 1128.0164 | 1157.77974 | 0 | 0 | 0 |
| Network 2 | -557.48334 | 4 | 7 | 535 | 1 | 1128.96668 | 1129.1792 | 1158.94255 | 1.16280778 | 1.16280778 | 1.16280778 |
| Network 3 | -557.6424 | 4 | 7 | 535 | 1 | 1129.2848 | 1129.49732 | 1159.26067 | 1.48092626 | 1.48092626 | 1.48092626 |
| Network 4 | -557.92218 | 4 | 7 | 535 | 1 | 1129.84437 | 1130.05689 | 1159.82024 | 2.04049583 | 2.04049583 | 2.04049583 |
| Network 5 | -578.93443 | 4 | 7 | 535 | 1 | 1171.86887 | 1172.08139 | 1201.84473 | 44.0649939 | 44.0649939 | 44.0649939 |
| Network 6 | -578.94751 | 4 | 7 | 535 | 1 | 1171.89503 | 1172.10755 | 1201.87089 | 44.0911535 | 44.0911535 | 44.0911535 |
| Network 7 | -578.96529 | 4 | 7 | 535 | 1 | 1171.93058 | 1172.1431 | 1201.90644 | 44.1267021 | 44.1267021 | 44.1267021 |
| Network 8 | -580.32144 | 4 | 7 | 535 | 1 | 1174.64288 | 1174.8554 | 1204.61875 | 46.8390073 | 46.8390073 | 46.8390073 |
| Network 9 | -580.32151 | 4 | 5 | 535 | 0 | 1170.64302 | 1170.75644 | 1192.05436 | 42.8391502 | 42.740048 | 34.2746167 |
| Network 10 | -580.32158 | 4 | 7 | 535 | 1 | 1174.64316 | 1174.85568 | 1204.61903 | 46.8392851 | 46.8392851 | 46.8392851 |

^a^Topology refers to each of the ten networks produced from the Phylonet analysis including Cypripedium × alaskanum, listed from highest (Network 1) to lowest (Network 10) total log probability (lnL).
^b^The number of parameters was estimated with p = (2n-3)+(2h), where p = number of parameters, n = number of tips, and h = number of hybridizations.
The AIC, AICc, BIC scores were calculated according to Yu et al. (2012).
Guide to deltaAIC and deltaAICc scores: less than 2 indicates there is substantial evidence to support the candidate model (i.e., the candidate model is almost as good as the best model), between 4 and 7 indicates that the candidate model has considerably less support, greater than 10 indicates that there is essentially no support for the candidate model (i.e., it is unlikely to be the best model). Guide for the deltaBIC scores: less than 2 is not worth more than a bare mention, between 2 and 6 indicates that the evidence against the candidate model is positive, between 6 and 10 indicates that the evidence against the candidate model is strong, greater than 10 indicates that the evidence is very strong.

Table S10: Calculation and comparison of 10 models produced by the PhyloNet analysis testing for up to one hybridization event in the network including Cypripedium × alaskanum.

| Model | LnL | params | d | e | j | x | AIC | AIC_wt | AICc | AICc_wt |
| --- | --- | --- | --- | --- | --- | --- | --- | --- | --- | --- |
| DEC | -129.59 | 3 | 0.024 | 0.008 | 0.000 | -0.27 | 265.185 | 0.298 | 265.647 | 0.319 |
| DEC+J | -174.85 | 4 | 0.024 | 0.008 | 0.000 | 0.000 | 357.690 | 0.000 | 358.475 | 0.000 |
| DIVALIKE | -129.75 | 3 | 0.026 | 0.003 | 0.000 | -0.27 | 265.492 | 0.256 | 265.953 | 0.274 |
| DIVALIKE+J | -128.21 | 4 | 0.023 | 0.000 | 0.018 | -0.26 | 264.419 | 0.437 | 265.204 | 0.399 |
| BAYAREALIKE | -134.94 | 3 | 0.027 | 0.071 | 0.000 | -0.96 | 275.878 | 0.001 | 276.340 | 0.002 |
| BAYAREALIKE+J | -132.34 | 4 | 0.025 | 0.064 | 0.008 | -1.35 | 272.680 | 0.007 | 273.464 | 0.006 |

Table S11: Results table of the BioGeoBEARS analysis using nine areas, showing the AIC and AICc scores and weights of each tested model for comparison.

Table S12: Results table of the BioGeoBEARS analysis using two areas (New and Old World), showing the AIC and AICc scores and weights of each tested model for comparison.

| Model | LnL | params | d | e | j | AIC | AIC_wt | AICc | AICc_wt |
| --- | --- | --- | --- | --- | --- | --- | --- | --- | --- |
| DEC | -38.84 | 2 | 0.020 | 0.003 | 0.000 | 81.677 | 0.000 | 81.903 | 0.000 |
| DEC+J | -32.81 | 3 | 0.006 | 0.000 | 0.074 | 71.628 | 0.016 | 72.090 | 0.016 |
| DIVALIKE | -36.61 | 2 | 0.023 | 0.001 | 0.000 | 77.227 | 0.001 | 77.454 | 0.001 |
| DIVALIKE+J | -33.37 | 3 | 0.010 | 0.000 | 0.058 | 72.740 | 0.009 | 73.201 | 0.009 |
| BAYAREALIKE | -49.54 | 2 | 0.010 | 0.010 | 0.000 | 103.074 | 0.000 | 103.300 | 0.000 |
| BAYAREALIKE+J | -28.70 | 3 | 0.002 | 0.000 | 0.079 | 63.391 | 0.974 | 63.852 | 0.974 |

# LITERATURE CITED

**Cai J, Liu X, Vanneste K, *et al.*** **2015**. The genome sequence of the orchid Phalaenopsis equestris. *Nature Genetics* **47**: 65–72.

**Chen SC, Liu ZJ, Chen LJ, Li LQ**. **2013**. *The Genus Cypripedium in China*. Peking: Science Press.

**Cribb P**. **1997**. *The Genus Cypripedium*. Portland: Timber Press.

**Eccarius W**. **2009**. *Orchideengattung Cypripedium*. EchinoMedia.

**Frosch W, Cribb P**. **2012**. *Hardy Cypripedium: Species, hybrids and cultivation*. Kew Publishing Kew.

**Hu C, Jiao Z, Deng X, *et al.*** **2022**. The ecological adaptation of the unparalleled plastome character evolution in slipper orchids. *Frontiers in Plant Science* **13**: 1075098.

**Lindley J**. **1840**. *The genera and species of orchidaceous plants*. London: Ridgways.

**Pfitzer EHH**. **1903**. *Orchidaceae–Pleonandrae*. Leipzig: Engelmann.

**Piet Q, Droc G, Marande W, *et al.*** **2022**. A chromosome-level, haplotype-phased Vanilla planifolia genome highlights the challenge of partial endoreplication for accurate whole-genome assembly. *Plant Communications* **3**: 100330.

**Yu Y, Degnan JH, Nakhleh L**. **2012**. The Probability of a Gene Tree Topology within a Phylogenetic Network with Applications to Hybridization Detection. *PLOS Genetics* **8**: e1002660.

**Zhang G-Q, Liu K-W, Li Z, *et al.*** **2017**. The *Apostasia* genome and the evolution of orchids. *Nature* **549**: 379–383.

**Zhang G-Q, Xu Q, Bian C, *et al.*** **2016**. The *Dendrobium catenatum* Lindl. genome sequence provides insights into polysaccharide synthase, floral development and adaptive evolution. *Scientific Reports* **6**: 19029.
