# Supplementary material for: Phylogenomic analysis of target enrichment and transcriptome data uncovers rapid radiation and extensive hybridization in the slipper orchid genus Cypripedium
Source: Ann Bot. 2024 Sep 12;134(7):1229–50. doi: 10.1093/aob/mcae161 (PMC11688532; doi:10.1093/aob/mcae161)
Supplement: mcae161_suppl_Supplementary_Figure_S2-S19 [file mcae161_suppl_supplementary_figure_s2-s19.docx]

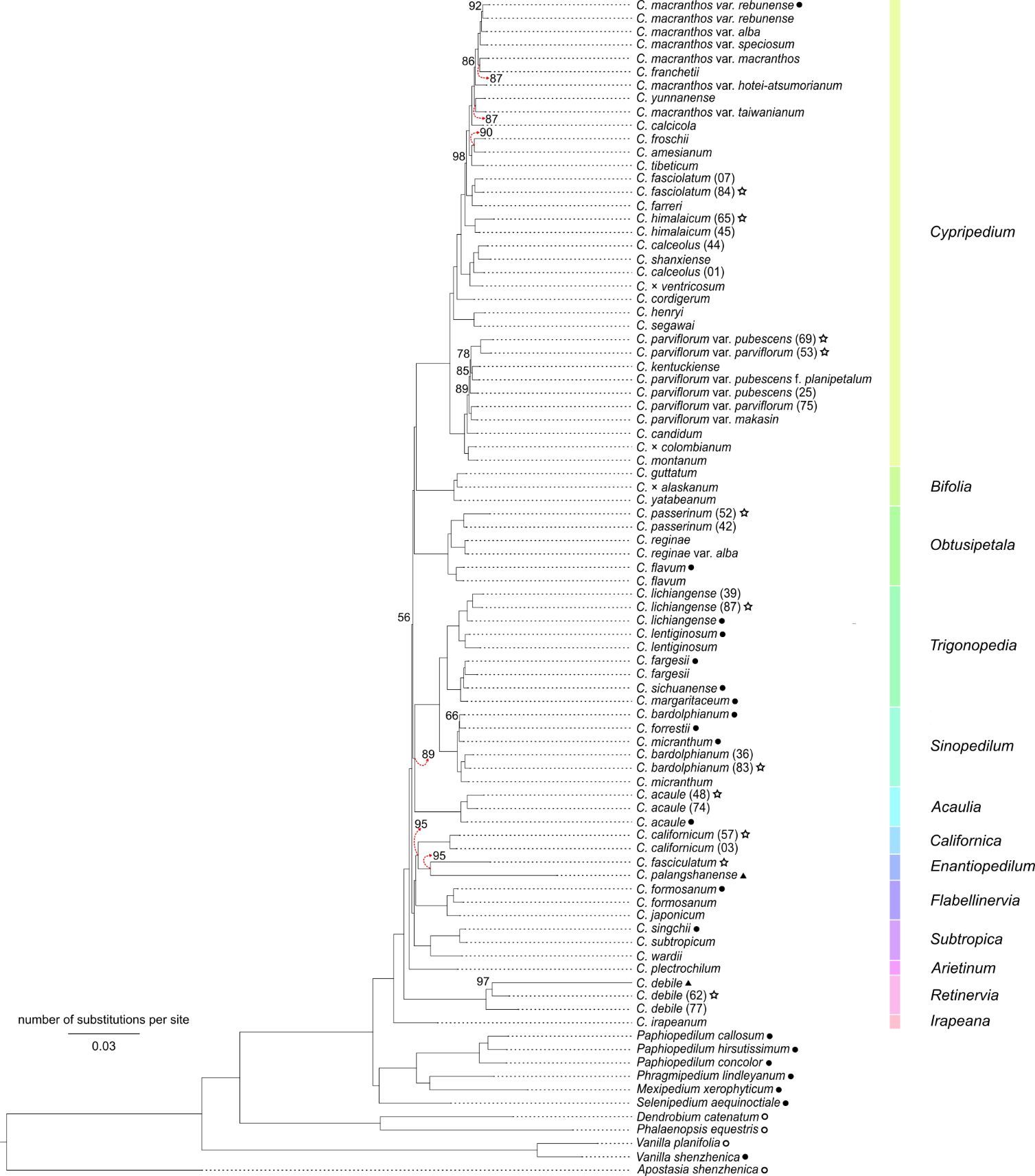


Figure S2: The concatenation-based phylogeny of *Cypripedium*, inferred with 913 nuclear loci using IQ-TREE. Bootstrap support values are shown above or below the branches when <100. Branches are annotated according to their section-level classification following Frosch and Cribb (2012). Tip symbols: filled circles “⬤” denote transcriptomes, unfilled circles “〇” denote genomes, filled triangles “▲” denote genome skimming sequences, and unfilled stars “☆” denote herbarium or old silica-dried specimens. Tips without symbols come from living specimens of the Botanical Collection at Oberhof.


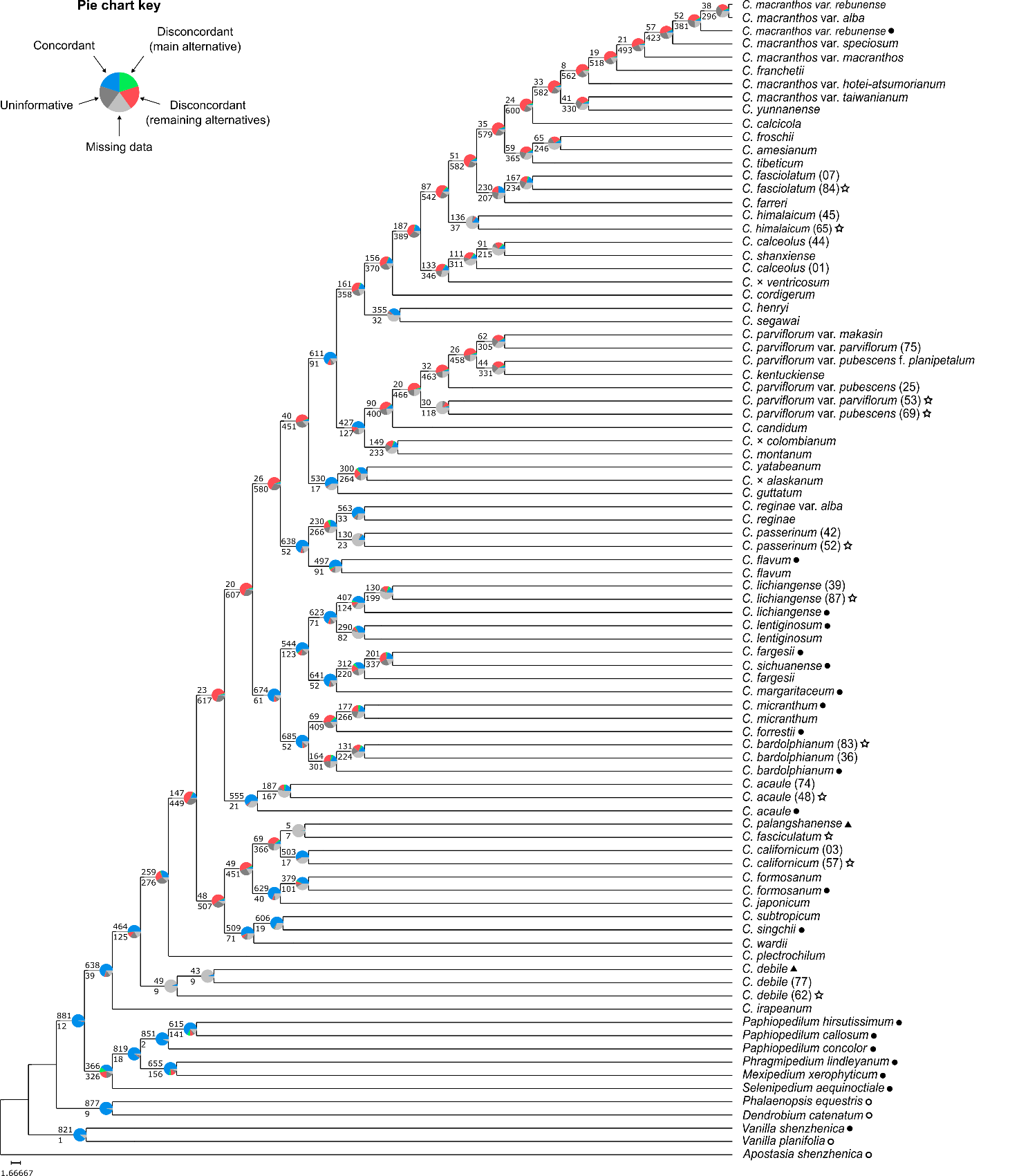


Figure S3: The output nuclear phylogeny of *Cypripedium* from the Phyparts analysis. The pie charts at the nodes indicate the proportions of informative concordant and discordant gene tree topologies (top and bottom numbers on each branch, respectively), along with the proportion of uninformative and missing loci (see pie chart key on the top left). Tip symbols: filled circles “⬤” denote transcriptomes, unfilled circles “〇” denote genomes, filled triangles “▲” denote genome skimming sequences, and unfilled stars “☆” denote herbarium or old silica-dried specimens. Tips without symbols come from living specimens of the Botanical Collection at Oberhof.


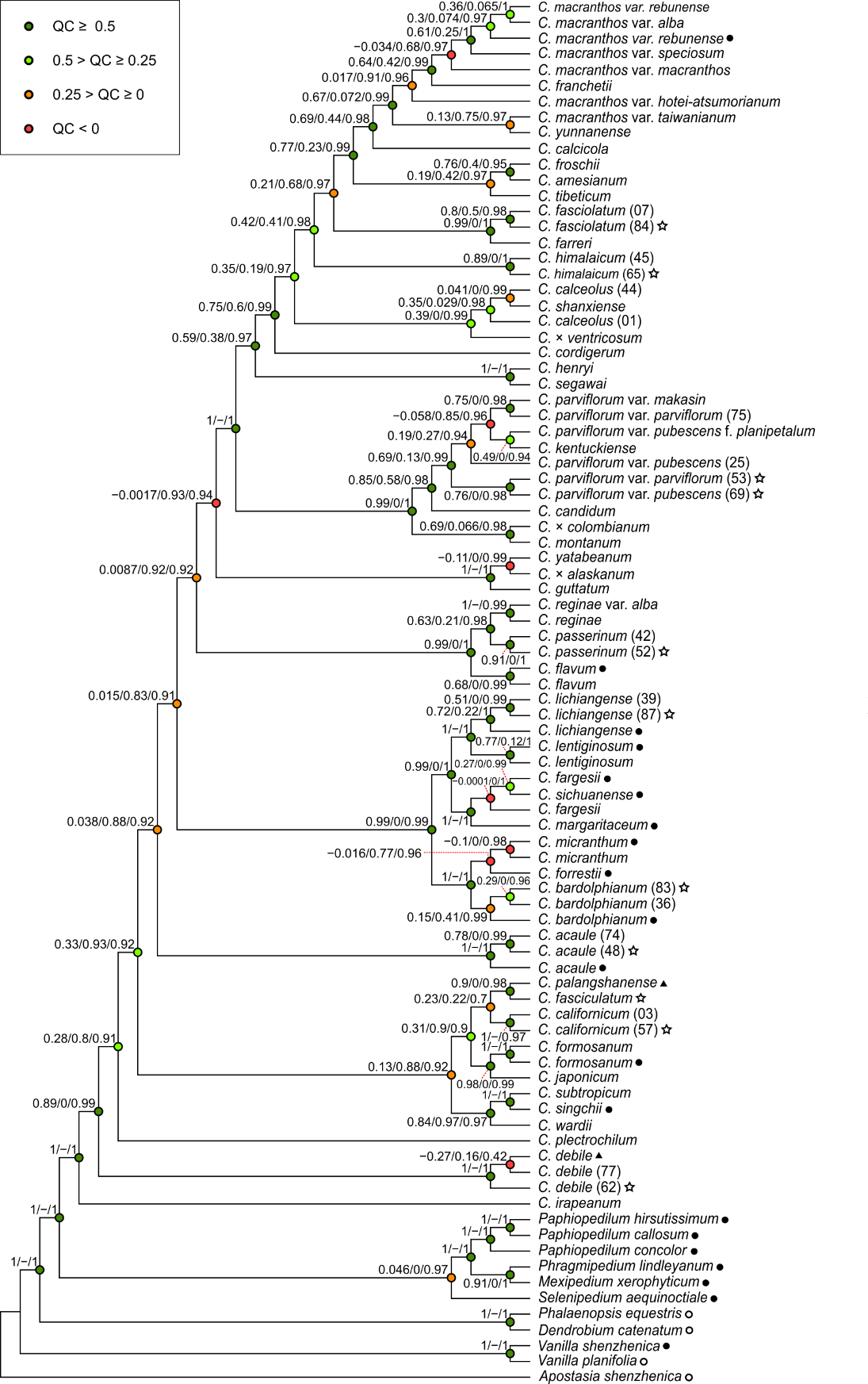


Figure S4: The output nuclear phylogeny of *Cypripedium* from the Quartet Sampling analysis. The nodes are annotated with the quartet support values Quartet Concordance/Quartet Differential/Quartet Informativeness in order (for interpretation, see Pease *et al.*, 2018) and colored based on the Quartet Concordance values (key to colors on the top left). Tip symbols: filled circles “⬤” denote transcriptomes, unfilled circles “〇” denote genomes, filled triangles “▲” denote genome skimming sequences, and unfilled stars “☆” denote herbarium or old silica-dried specimens. Tips without symbols come from living specimens of the Botanical Collection at Oberhof.


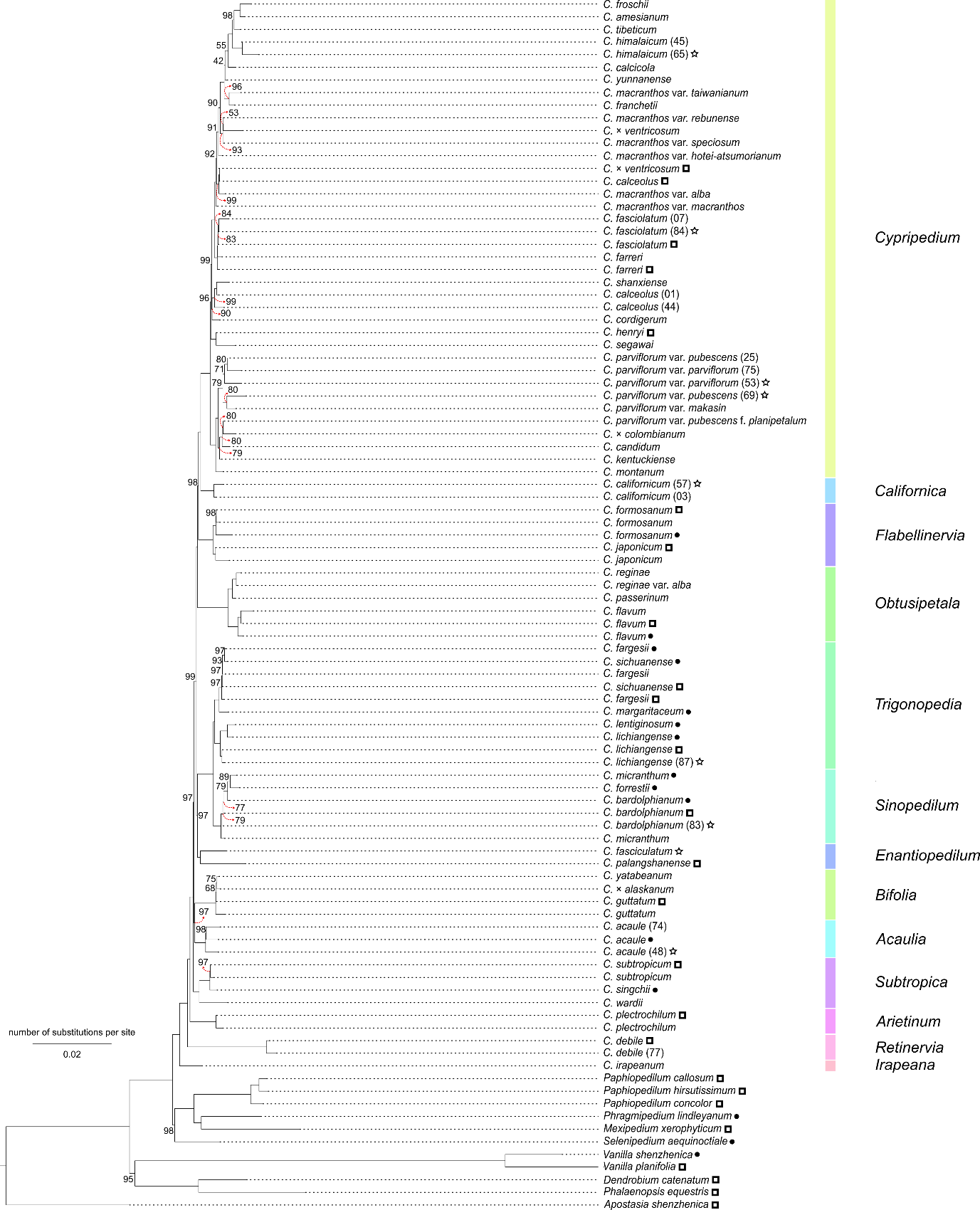


Figure S5: The concatenation-based phylogeny of *Cypripedium*, inferred with 80 chloroplast loci using IQ-TREE. Bootstrap support values are shown above or below the branches when <100. Branches are annotated according to their section-level classification following Frosch and Cribb (2012). Tip symbols: filled circles “⬤” denote transcriptomes, unfilled squares “◻” denote chloroplast genomes, filled triangles “▲” denote genome skimming sequences, and unfilled stars “☆” denote herbarium or old silica-dried specimens. Tips without symbols come from living specimens of the Botanical Collection at Oberhof.


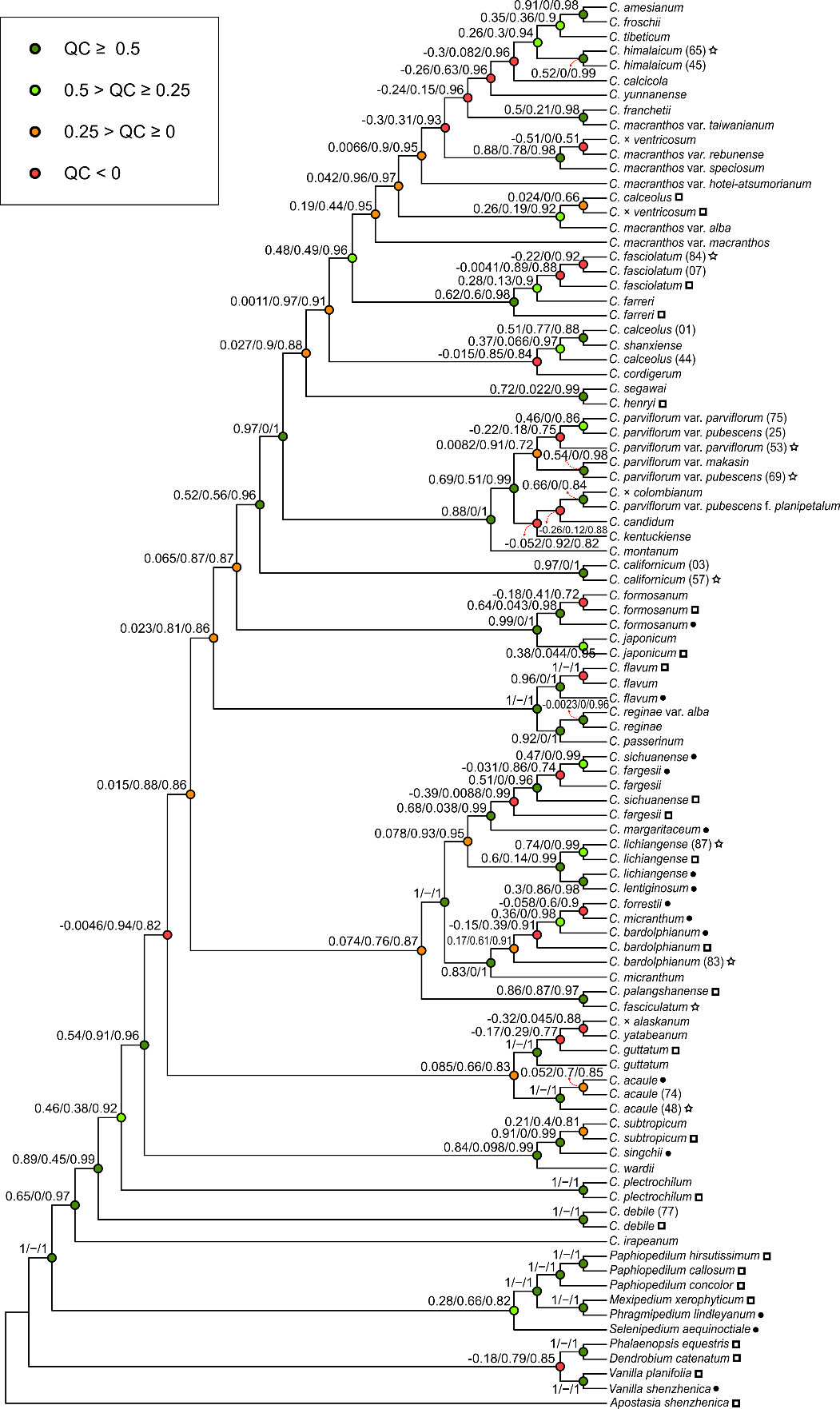


Figure S6: The output chloroplast *Cypripedium* phylogeny from the Quartet Sampling analysis. The nodes are annotated with the quartet support values Quartet Concordance/Quartet Differential/Quartet Informativeness in order (for interpretation, see Pease *et al.*, 2018) and colored based on the Quartet Concordance values (key to colors on the top left). Tip symbols: filled circles “⬤” denote transcriptomes, unfilled squares “◻” denote chloroplast genomes, filled triangles “▲” denote genome skimming sequences, and unfilled stars “☆” denote herbarium or old silica-dried specimens. Tips without symbols come from living specimens of the Botanical Collection at Oberhof.


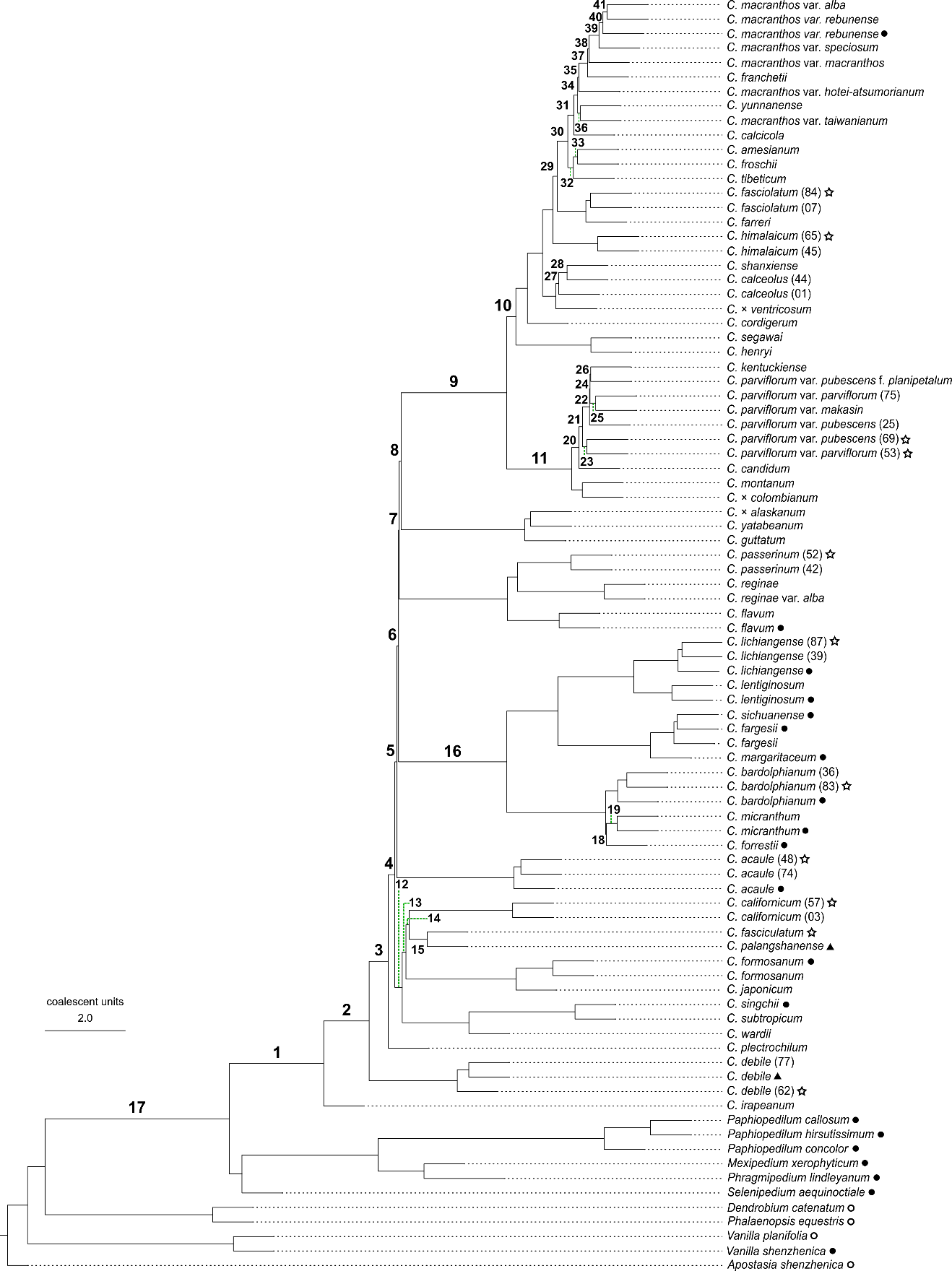


Figure S7: The nuclear ASTRAL phylogeny of Cypripedium annotated with the corresponding branch numbers referred to in Supplementary Table S9 for the results of the anomaly zone test calculations and in Figure 3 B. Tip symbols: filled circles “⬤” denote transcriptomes, unfilled circles “〇” denote genomes, filled triangles “▲” denote genome skimming sequences, and unfilled stars “☆” denote herbarium or old silica-dried specimens. Tips without symbols come from living specimens of the Botanical Collection at Oberhof.


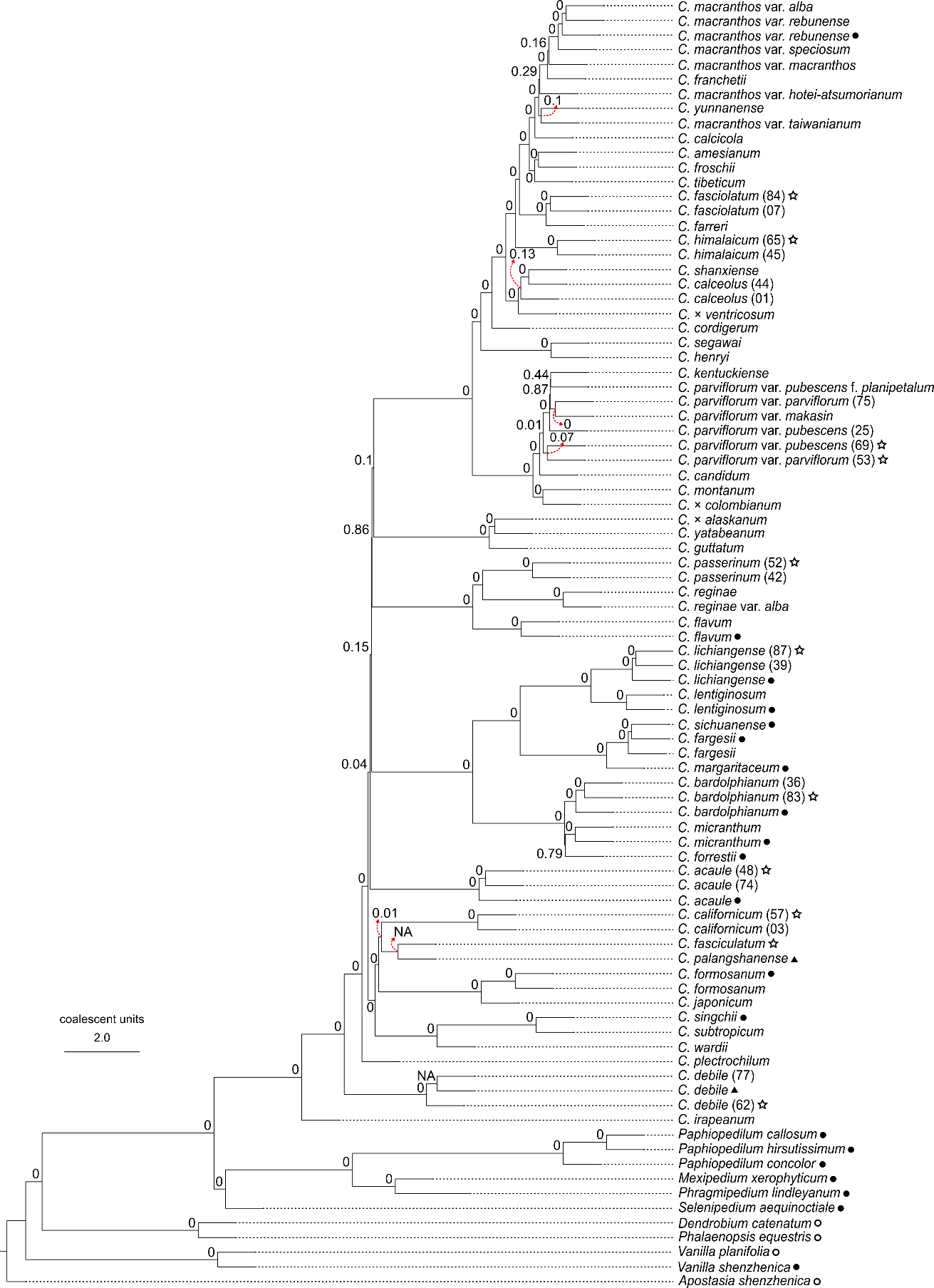


Figure S8: Results of the polytomy test on the nuclear ASTRAL phylogeny of *Cypripedium*. Tip symbols: filled circles “⬤” denote transcriptomes, unfilled circles “〇” denote genomes, filled triangles “▲” denote genome skimming sequences, and unfilled stars “☆” denote herbarium or old silica-dried specimens. Tips without symbols come from living specimens of the Botanical Collection at Oberhof.


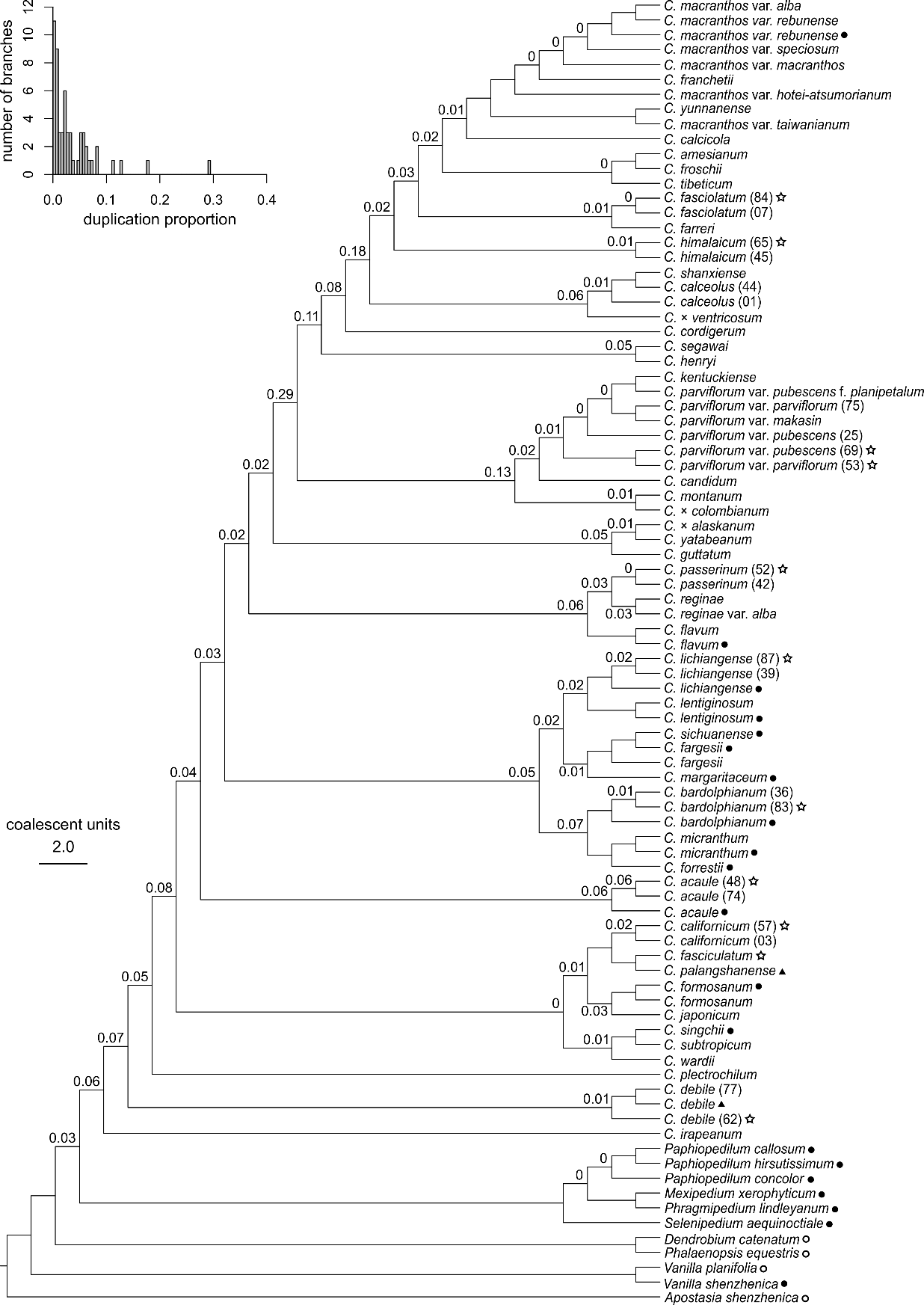


Figure S9: Results of the gene duplication test based on the subclade orthogroup tree topology method implemented with the nuclear ASTRAL phylogeny of *Cypripedium*, using the bootstrap filtering approach. The proportions of duplicated genes are labeled above or below the phylogeny’s branches. The number of branches is plotted against the gene duplication proportions on the top left. Branches that did not receive a label did not meet the filtering requirements. Tip symbols: filled circles “⬤” denote transcriptomes, unfilled circles “〇” denote genomes, filled triangles “▲” denote genome skimming sequences, and unfilled stars “☆” denote herbarium or old silica-dried specimens. Tips without symbols come from living specimens of the Botanical Collection at Oberhof.


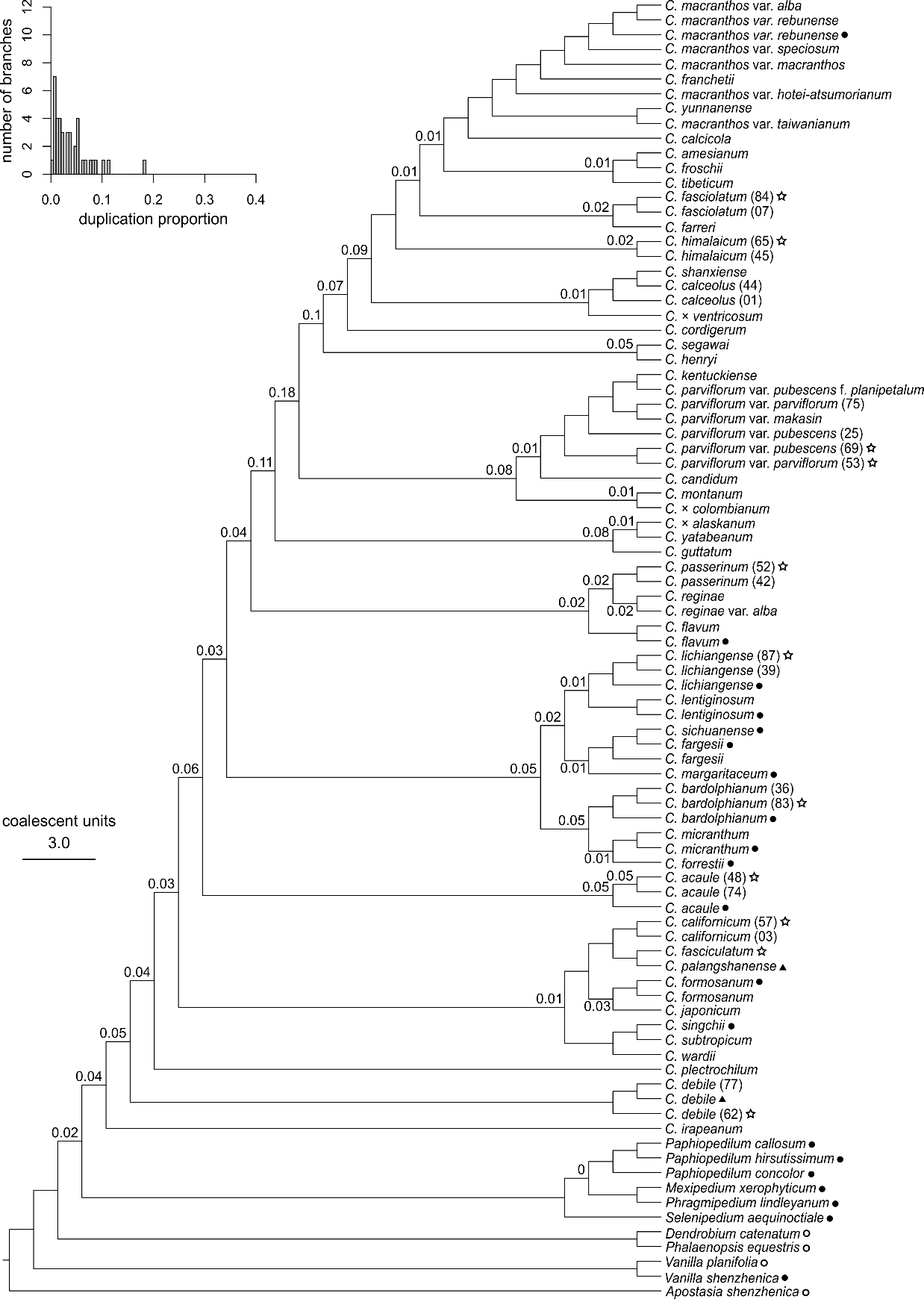


Figure S10: Results of the gene duplication test based on the subclade orthogroup tree topology method implemented with the nuclear ASTRAL phylogeny of *Cypripedium*, using the local topology filtering approach. The proportions of duplicated genes are labeled above or below the phylogeny’s branches. The number of branches is plotted against the gene duplication proportions on the top left. Branches that did not receive a label did not meet the filtering requirements. Tip symbols: filled circles “⬤” denote transcriptomes, unfilled circles “〇” denote genomes, filled triangles “▲” denote genome skimming sequences, and unfilled stars “☆” denote herbarium or old silica-dried specimens. Tips without symbols come from living specimens of the Botanical Collection at Oberhof.


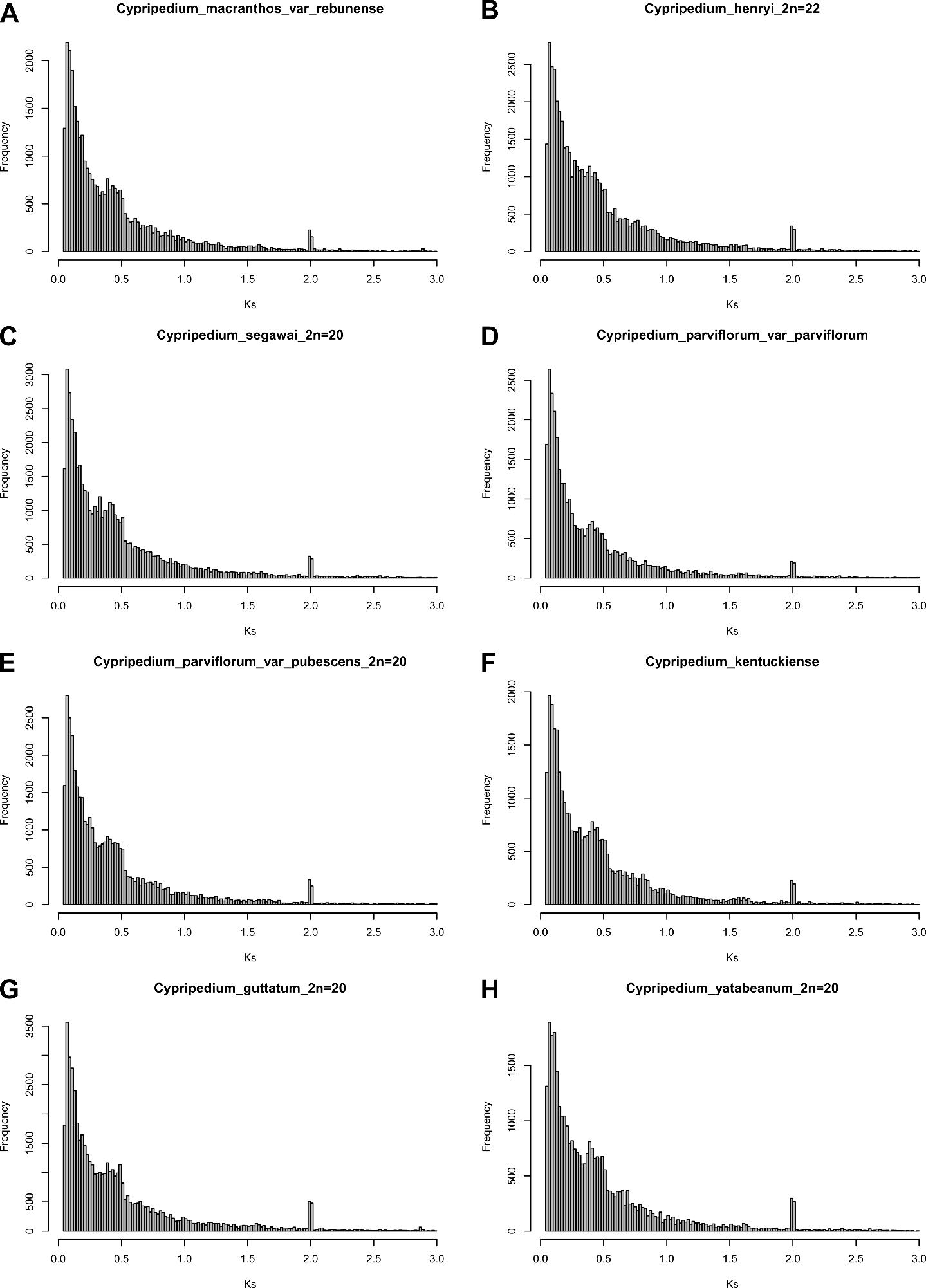

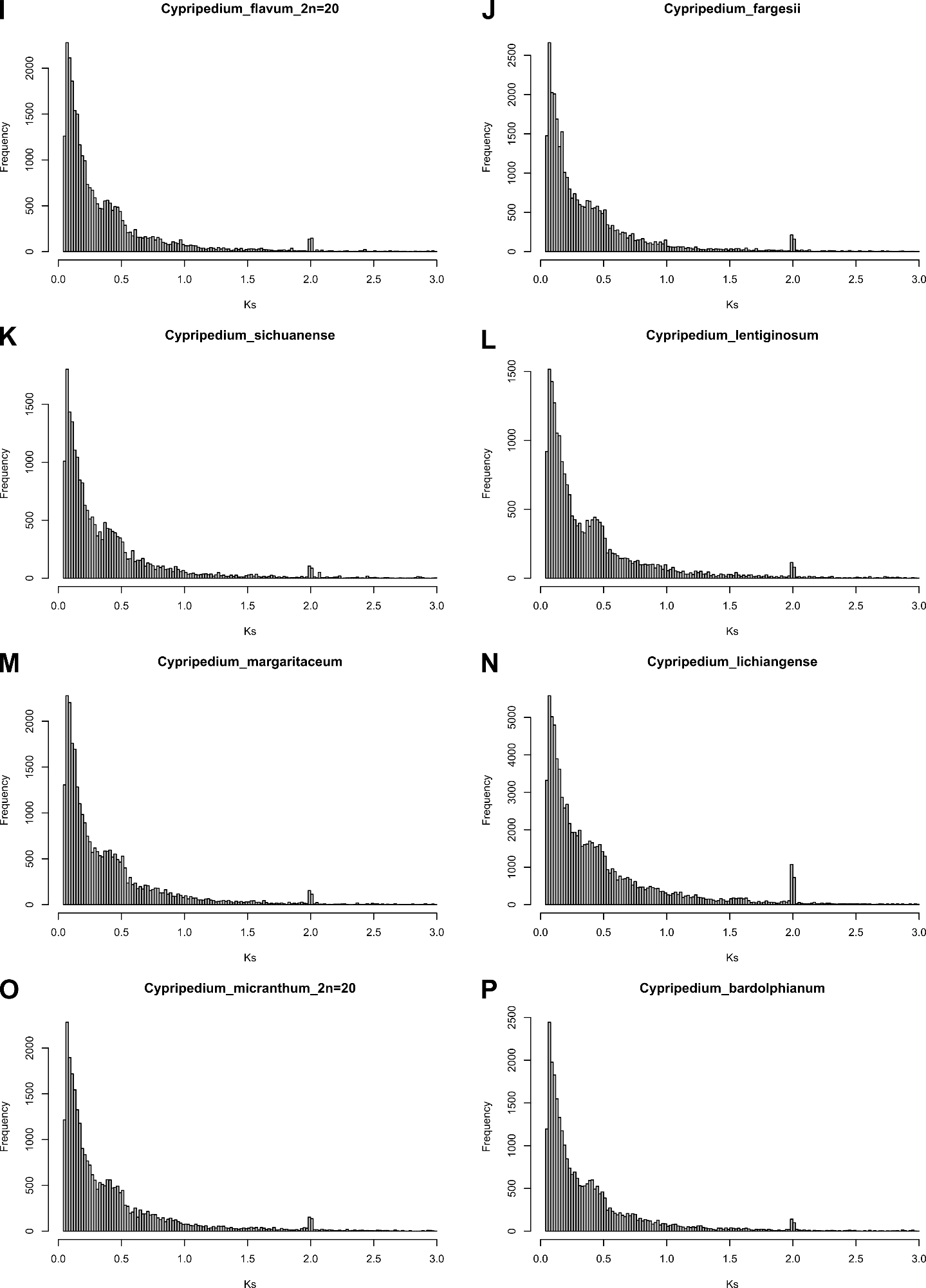

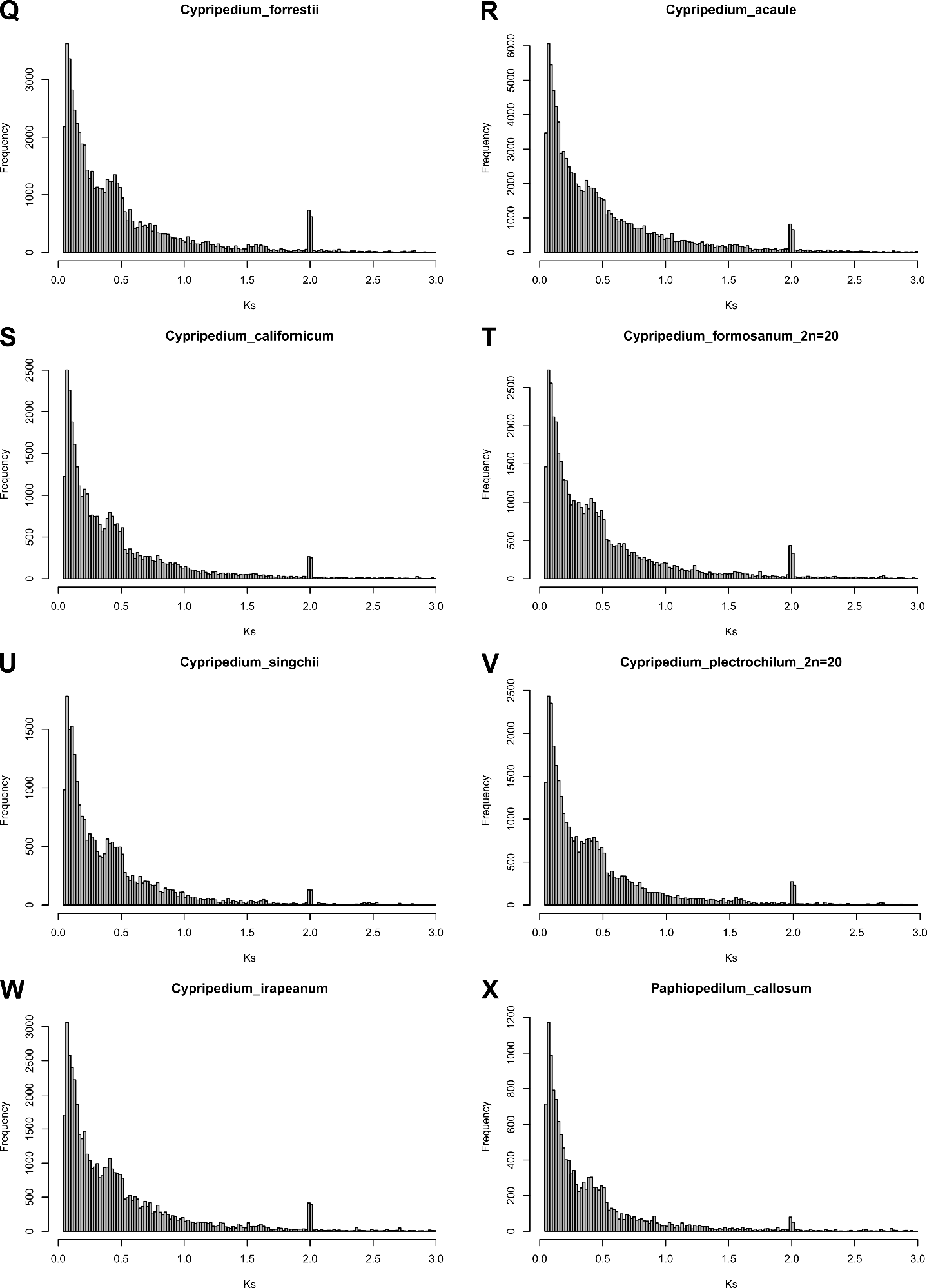

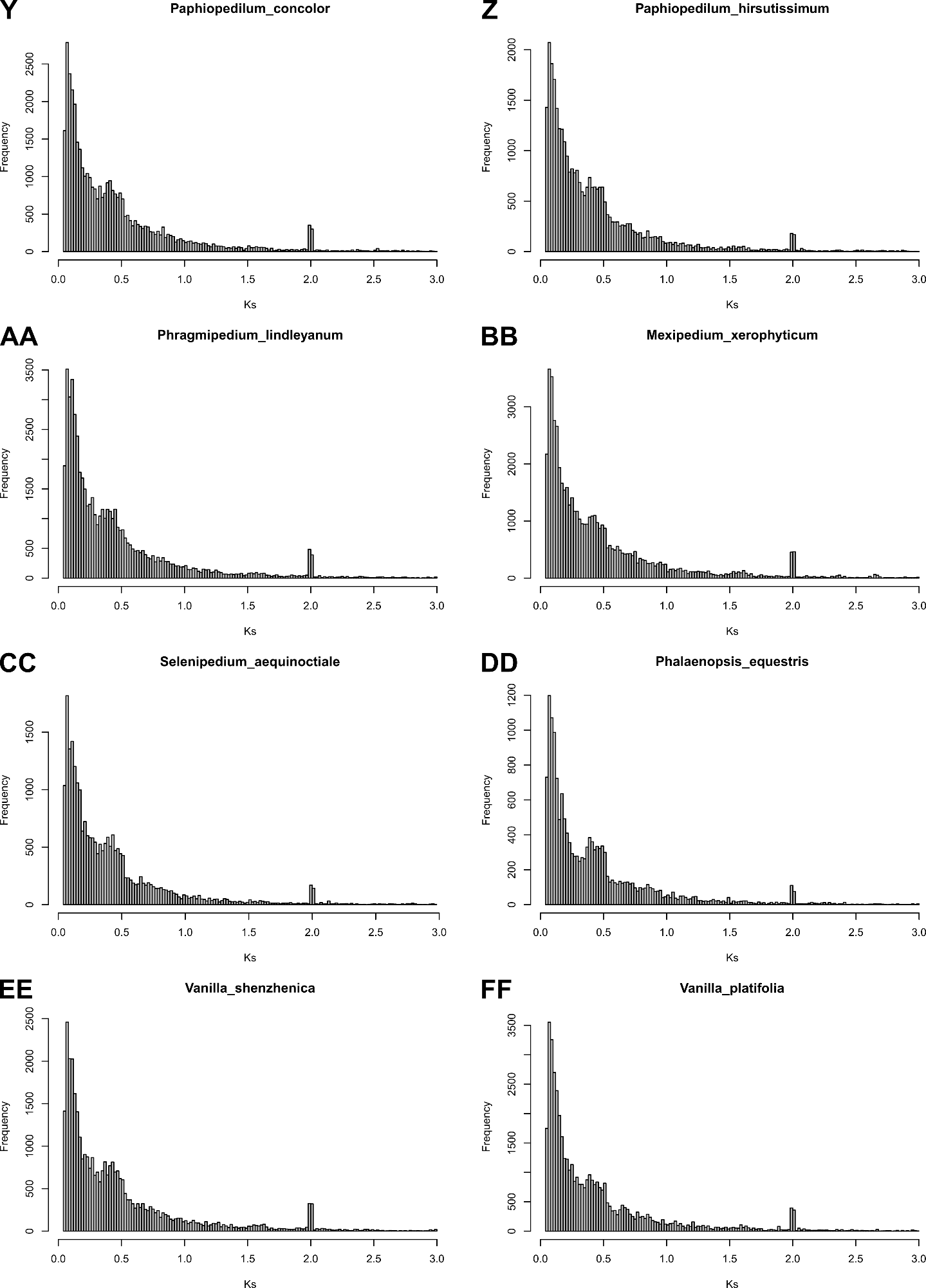

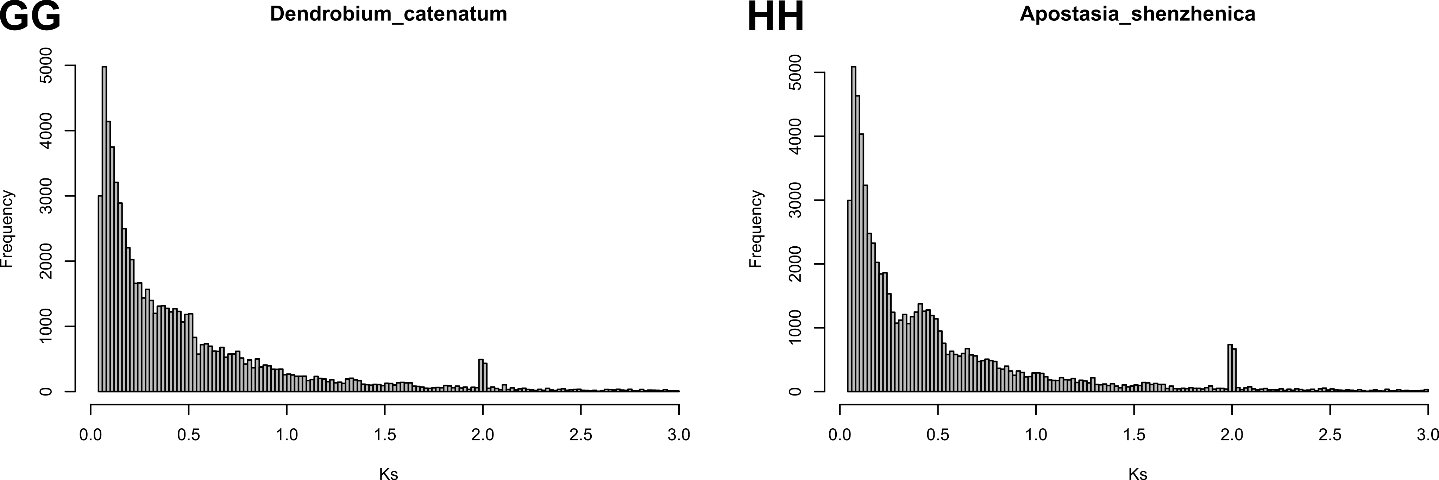


Figure S11: Ks plots for the *Cypripedium* transcriptomes generated in this study and additional orchid transcriptomes obtained from the NCBI (see Supplementary Tables S4 and S6). The usual count of diploid chromosomes is given for some *Cypripedium* species in the figure’s title.


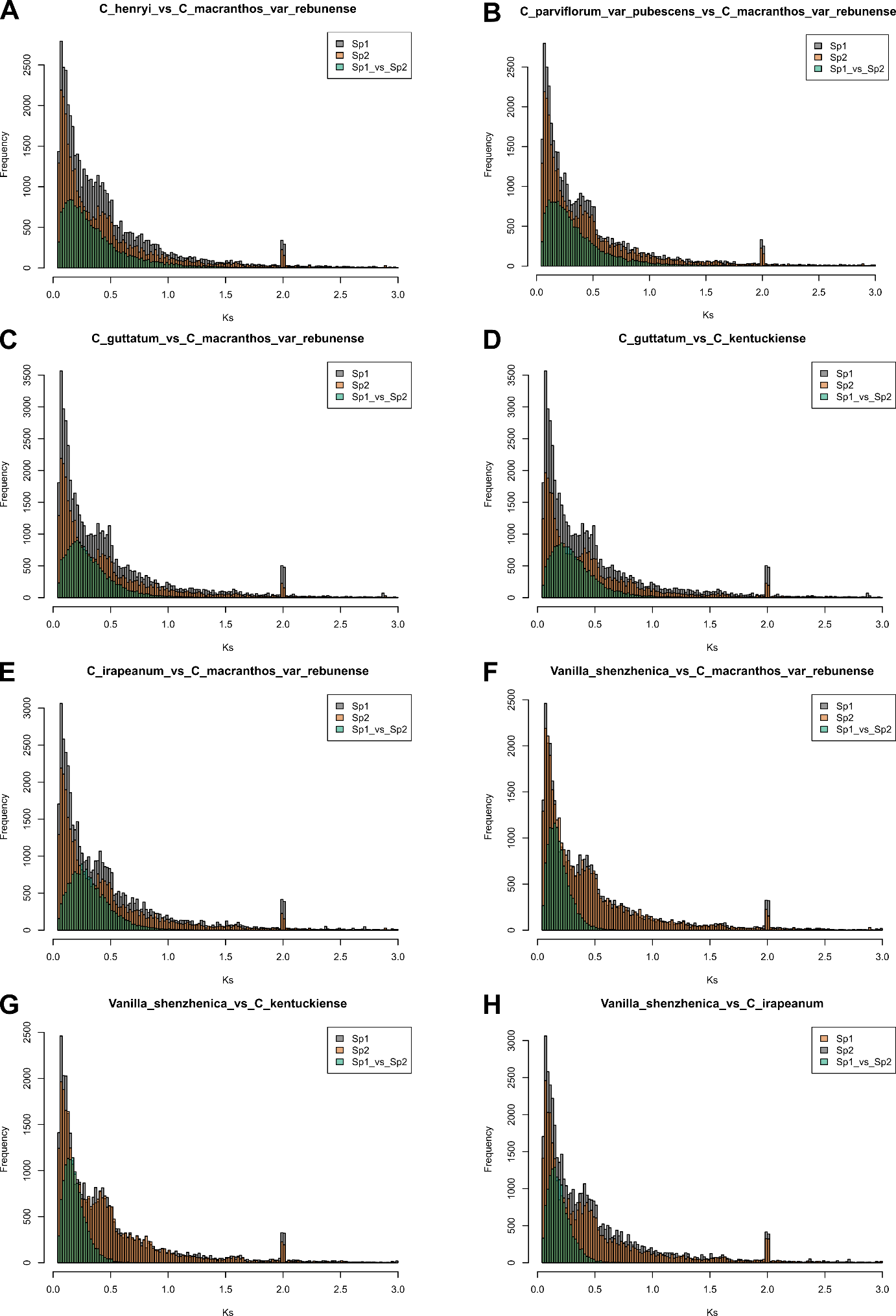

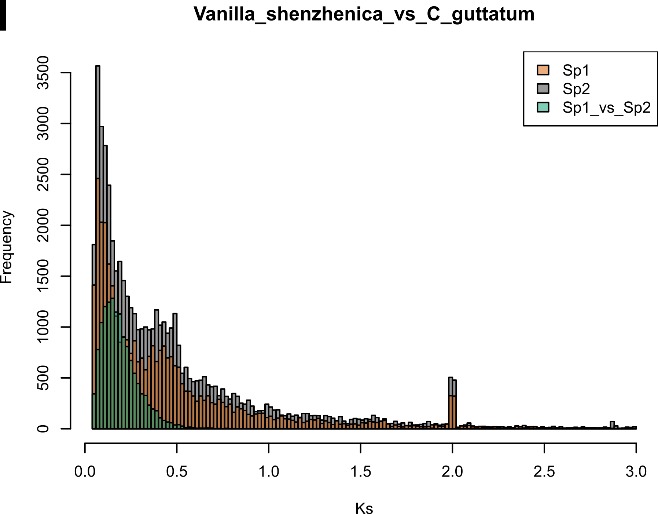


Figure S12: Ks plots comparison between species of the genus *Cypripedium* or between species of the genus *Cypripedium* and other orchids (see Supplementary Tables S4 and S6 for sequence details).


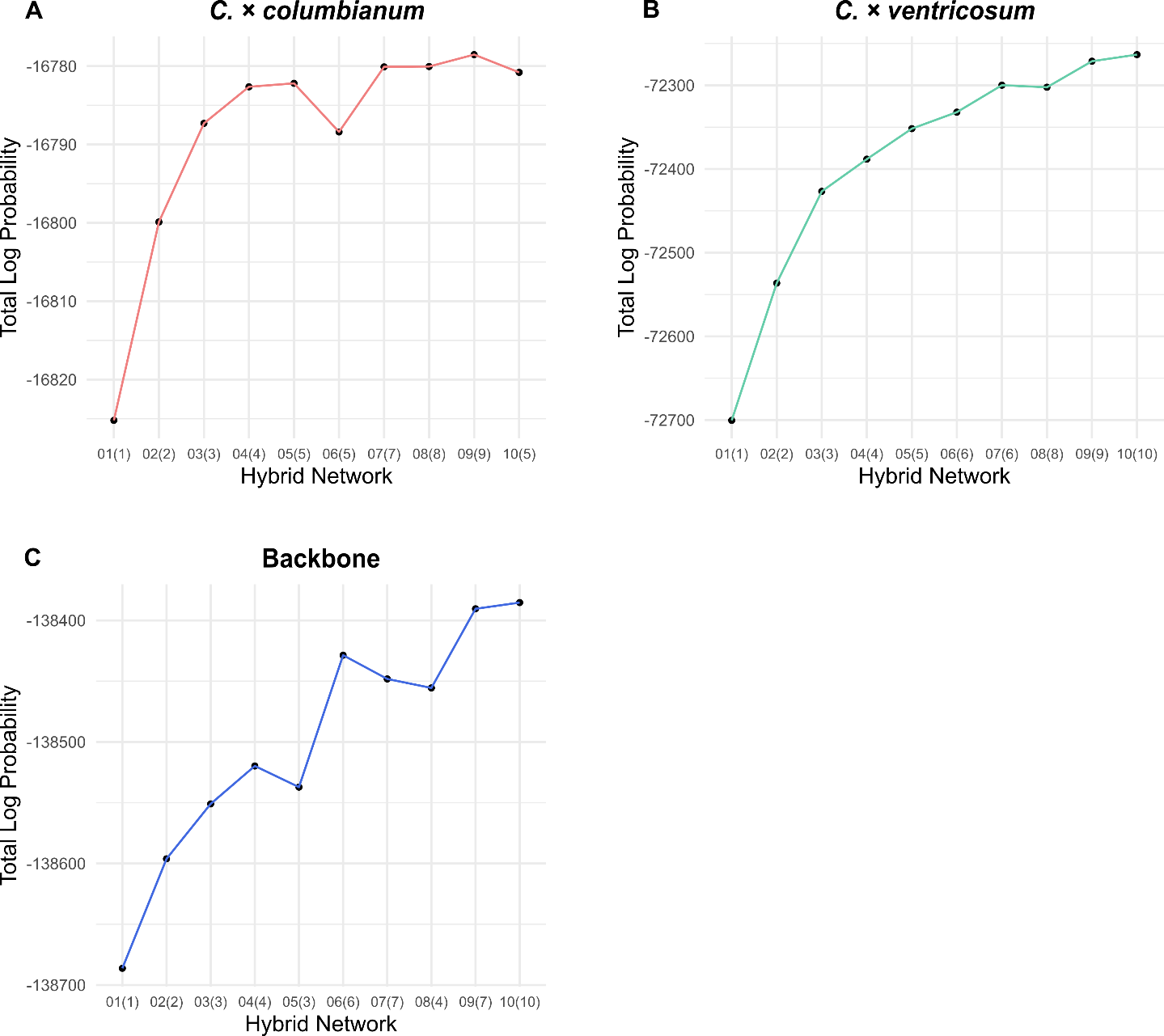


Figure S13: The total log probabilities (y-axis) of the most likely network from each run for the tests including (A) *C. × columbianum*, (B) *C. × ventricosum,* and (C) taxa from all sections to test for hybridizations at the backbone. Network IDs (x-axis): maximum number of hybridization events tested followed by the actual number of produced hybridization events in the parenthesis.


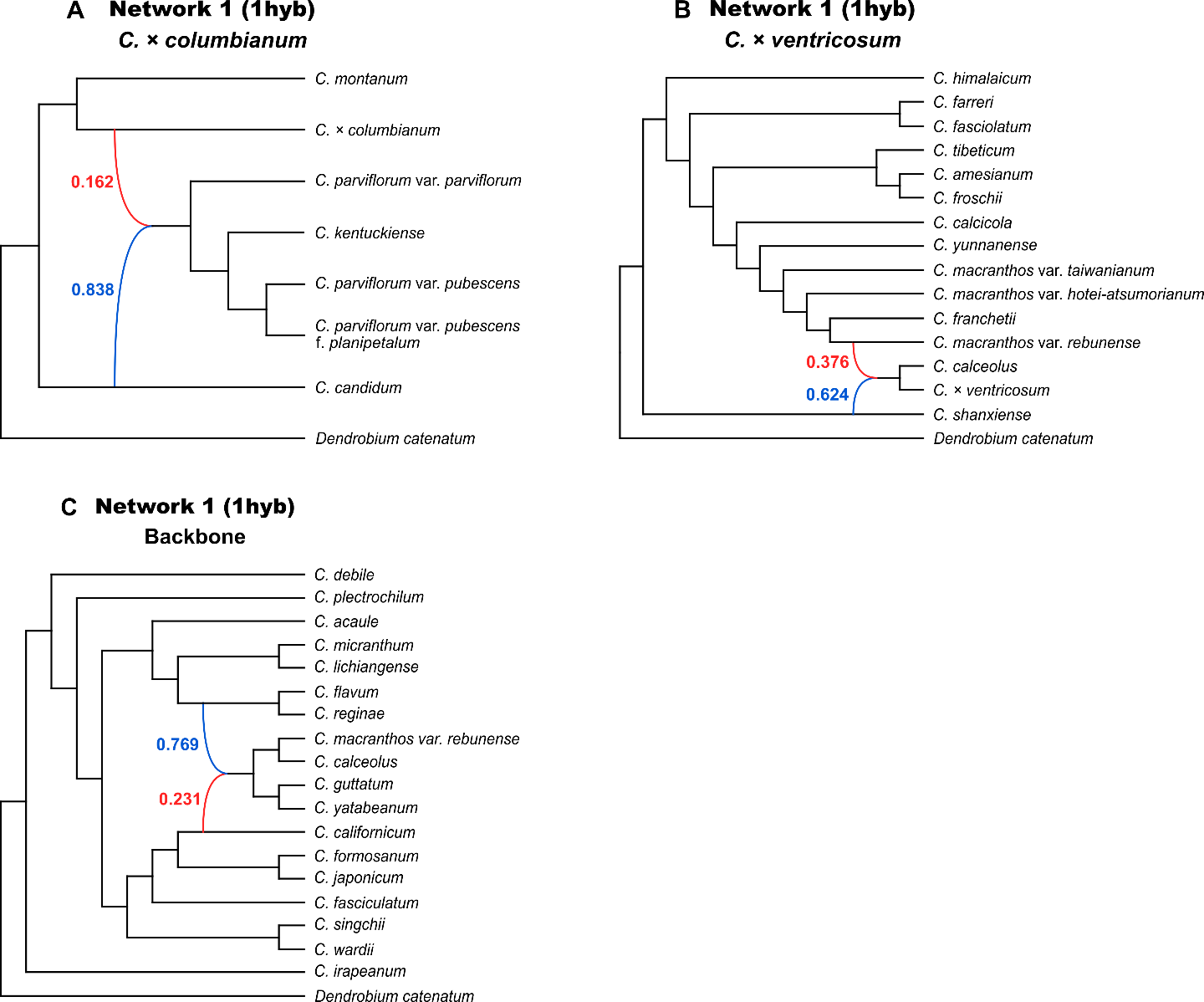


Figure S14: Phylogenetic networks with the highest total log probabilities resulting from the PhyloNet analysis testing for one hybridization event for the extracted subclades with the hybrids (A) *C. × columbianum* and (B) *C. × ventricosum* or (C) subclades from all sections to test for reticulation in the backbone. The inheritance probabilities are shown for each parent hybrid edge (blue = major hybrid edge; red = minor hybrid edge).


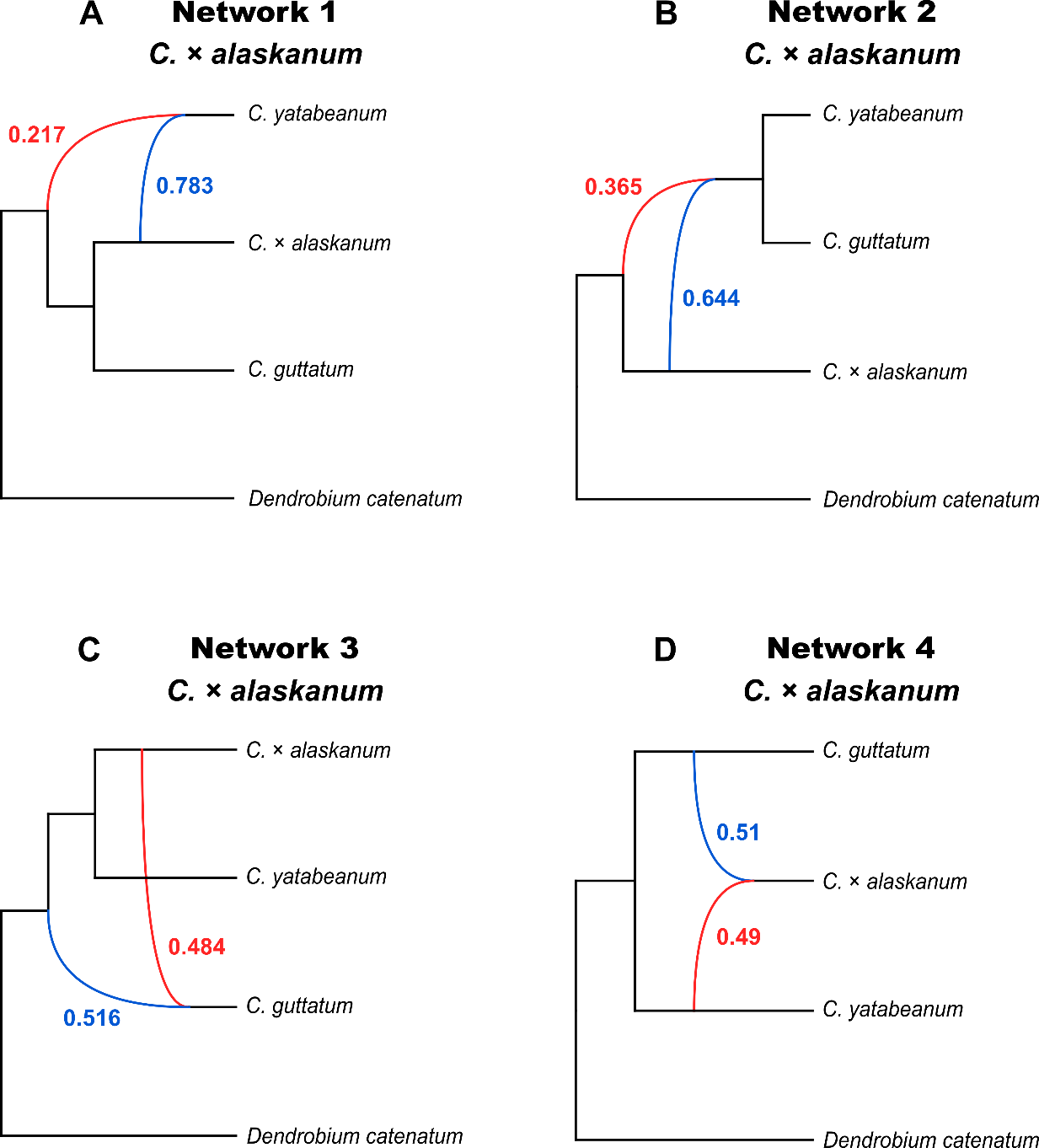


Figure S15: Phylogenetic networks including *C. × alaskanum* testing for one hybridization event (using the -po parameter) with the (A) first, (B) second, (C) third, and (D) fourth highest total log probability values. Based on the deltaAIC, deltaAICc, and deltaBIC scores listed in Supplementary Table S10, there is substantial evidence to support that the candidate models (B) and (C) are almost as good as the best model (A) (i.e., deltaAIC, deltaAICc, and deltaBIC < 2), while the support for candidate model (C) is comparatively lower (i.e., deltaAIC, deltaAICc, and deltaBIC = 2.04)


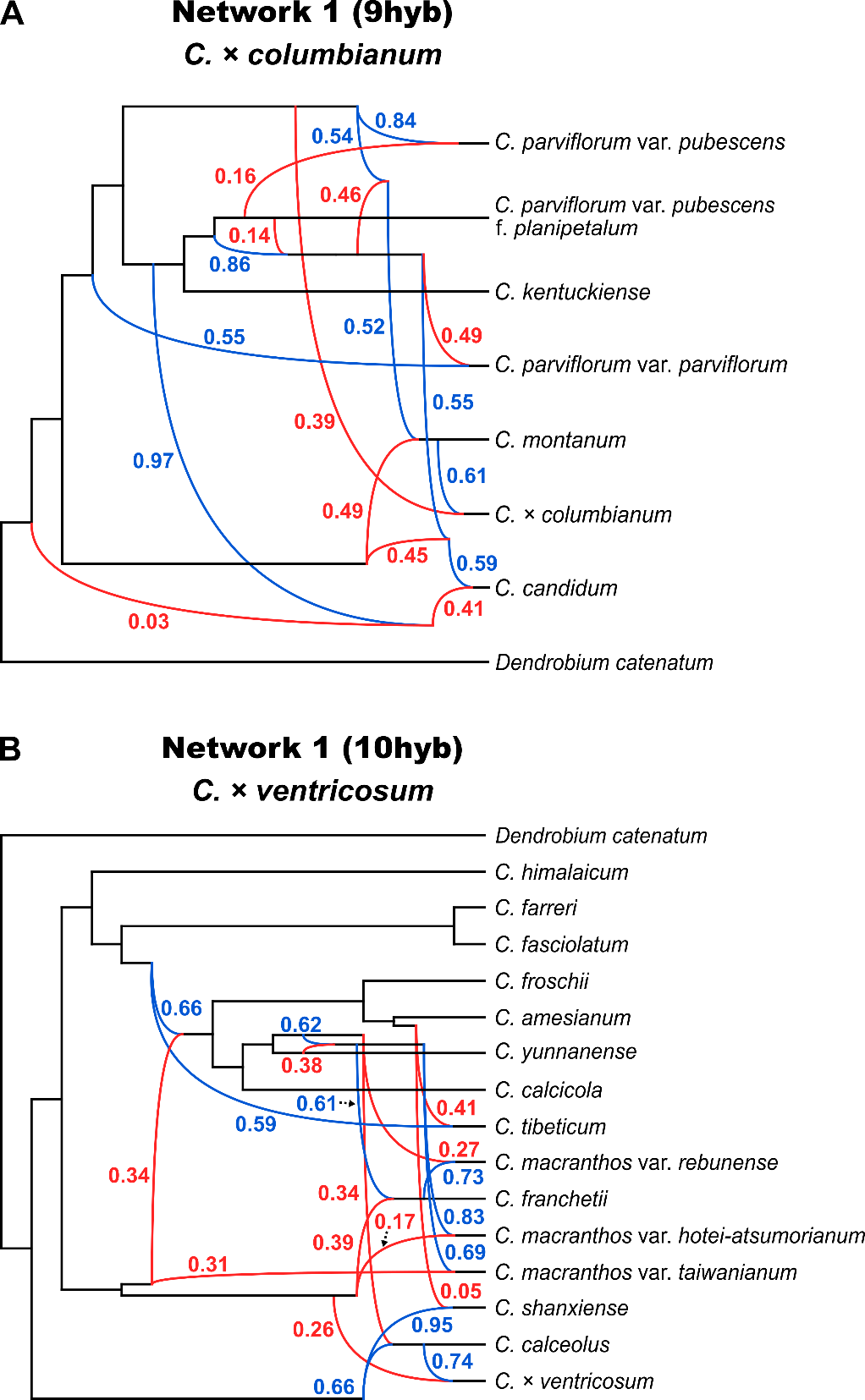


Figure S16: Phylogenetic networks with the overall highest total log probabilities testing for nine and ten hybridization events for the extracted subclades with the hybrids (A) *C. × columbianum* and (B) *C. × ventricosum*, respectively. The inheritance probabilities are shown for each parent hybrid edge (blue = major hybrid edge; red = minor hybrid edge).


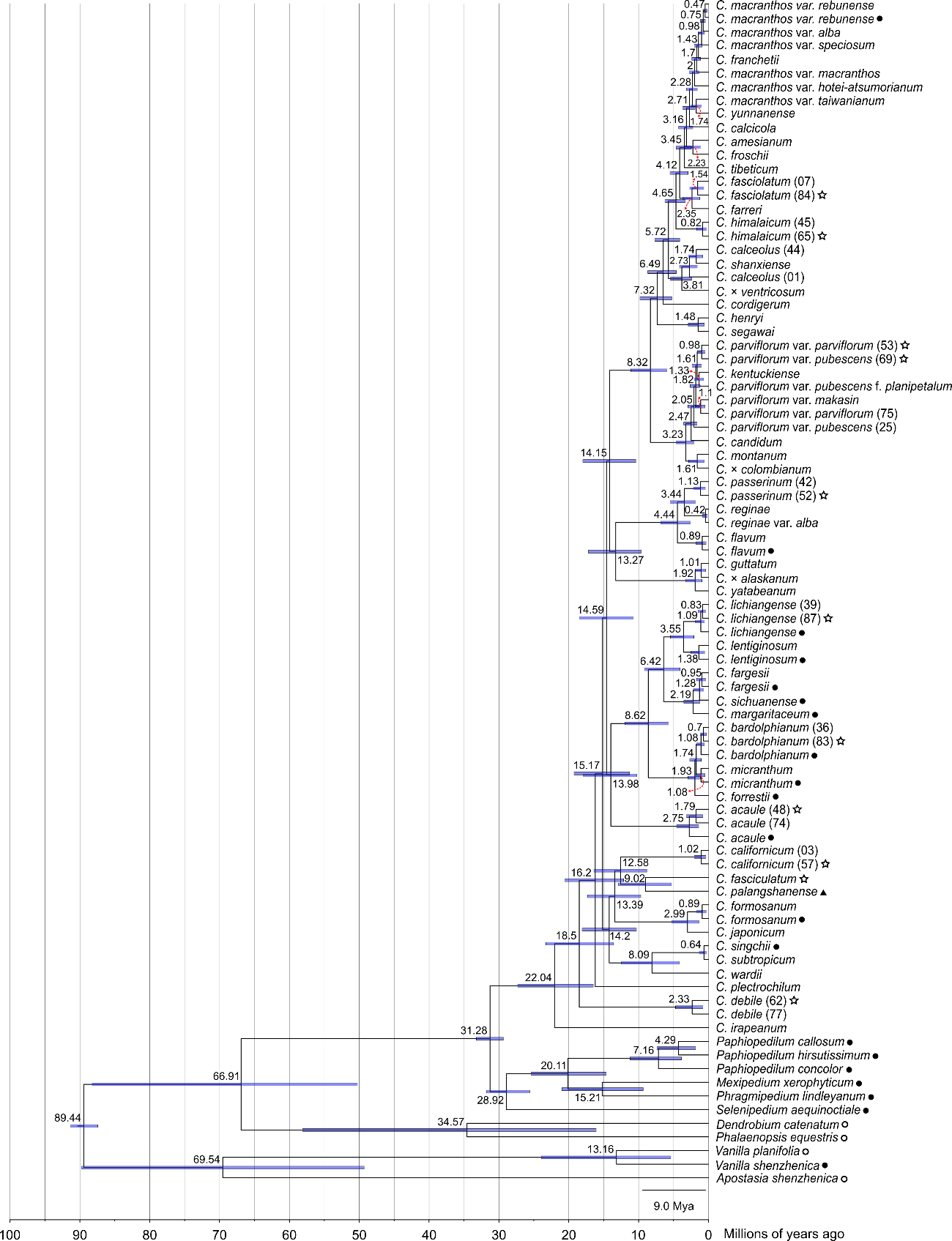

Figure S17: The maximum clade credibility tree of *Cypripedium* resulting from the molecular dating analysis with BEAST 2. The posterior median node height estimates (in Ma) are shown on the nodes, together with the 95% HPD bars. A time scale with the time before the present is shown at the bottom. Tip symbols: filled circles “⬤” denote transcriptomes, unfilled circles “〇” denote genomes, filled triangles “▲” denote genome skimming sequences, and unfilled stars “☆” denote herbarium or old silica-dried specimens. Tips without symbols come from living specimens of the Botanical Collection at Oberhof.


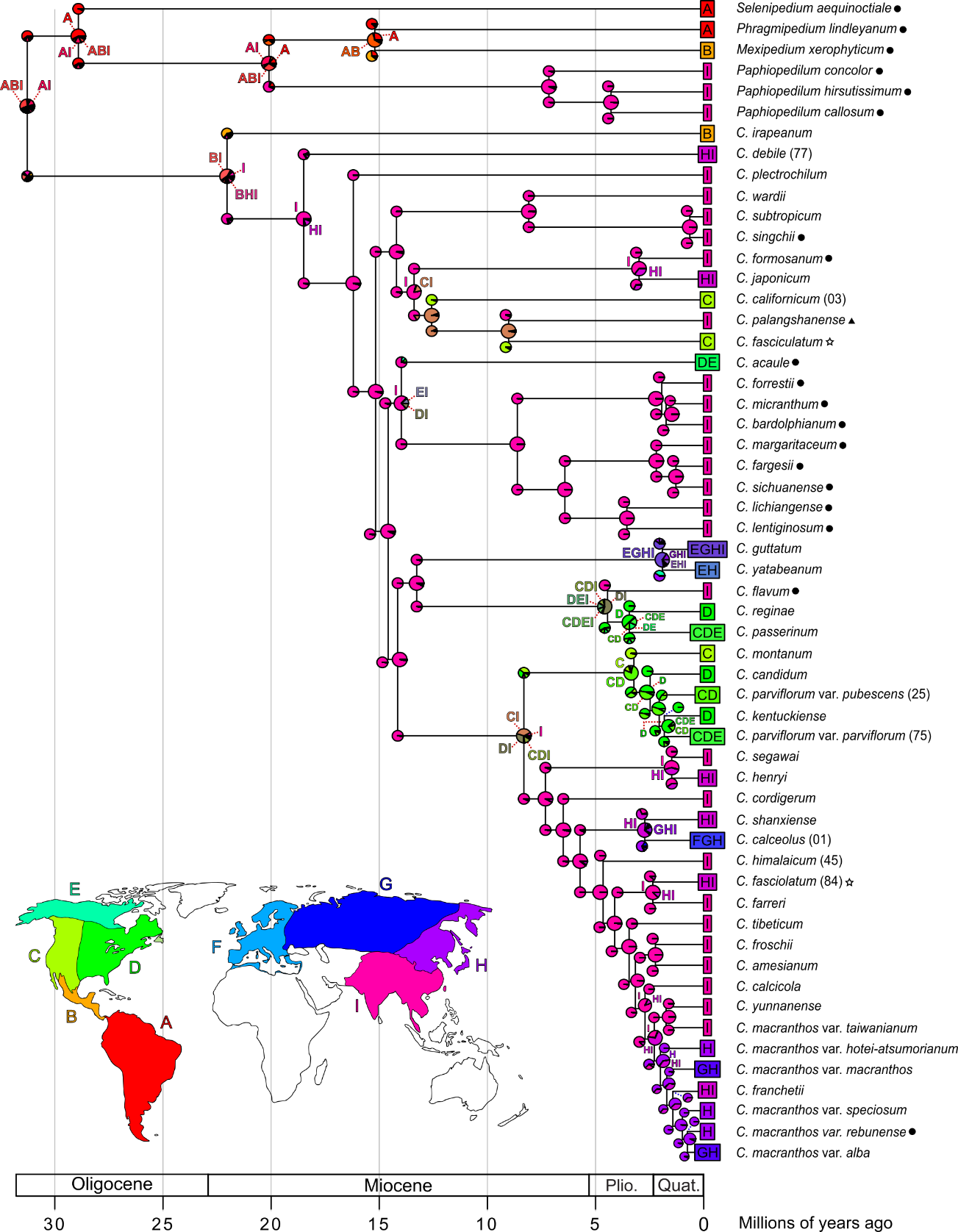


Figure S18: The relative proportions of the ancestral ranges estimated by BioGeoBEARS (nine area test, DEC model) and plotted as pie charts on the corresponding nodes of the dated maximum clade credibility tree of slipper orchids. Key to area codes: A = South America; B = Central America and Mexico; C = Western North America; D = Eastern North America; E = Northern North America; F = Eastern and Central Europe, the Mediterranean, and Scandinavia; G = Eastern Europe and Eurasia; H = Eastern Russia and Northeast Asia; I = Southeast Asia. Plio. = Pliocene; Quat. = Quaternary. Tip symbols: filled circles “⬤” denote transcriptomes, filled triangles “▲” denote genome skimming sequences, and unfilled stars “☆” denote herbarium or old silica-dried specimens. Tips without symbols come from living specimens of the Botanical Collection at Oberhof.


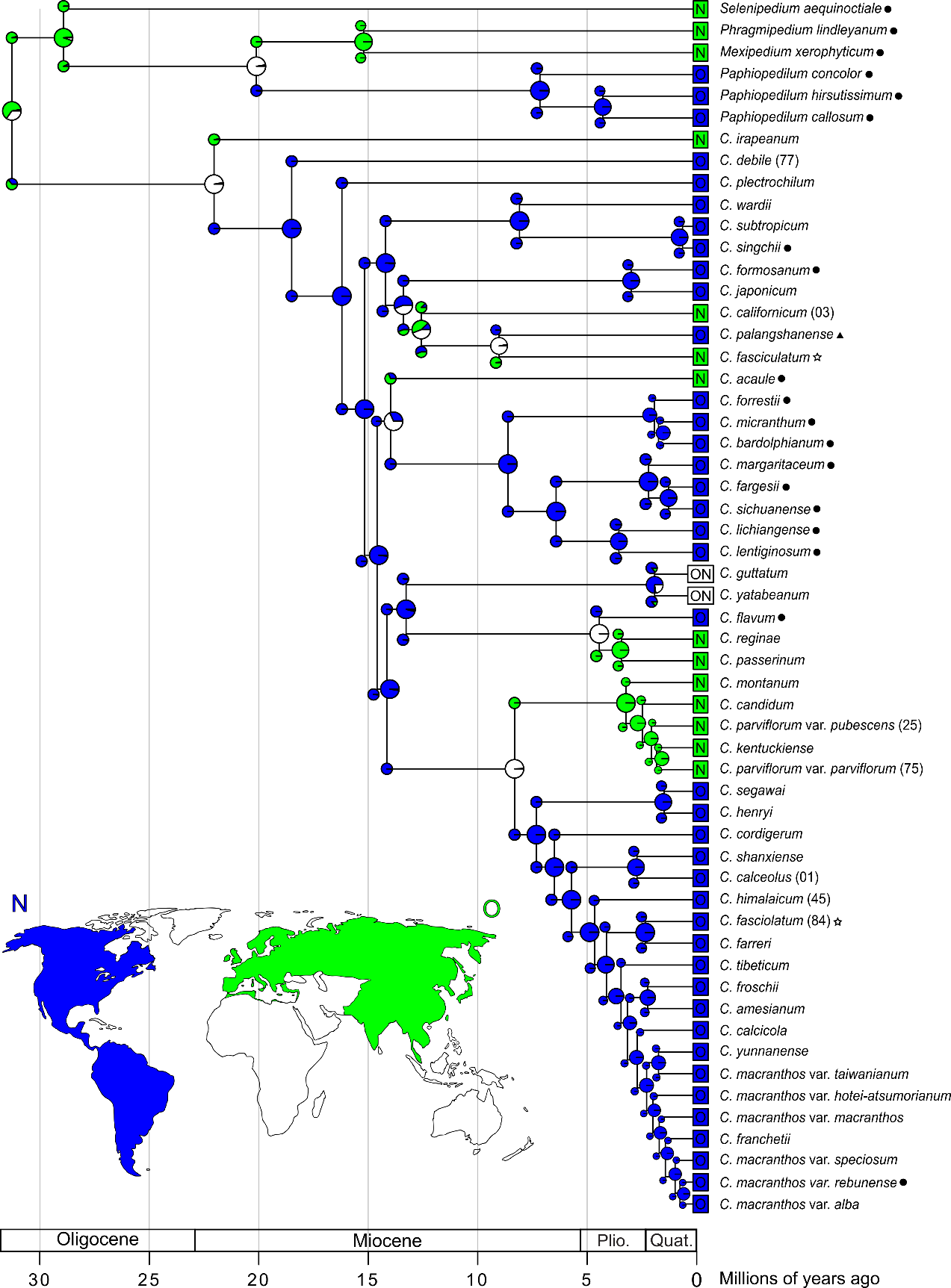


Figure S19: The relative proportions of the ancestral ranges estimated by BioGeoBEARS (two-area test, DIVALIKE model) and plotted as pie charts on the corresponding nodes of the dated maximum clade credibility tree of slipper orchids. Key to area codes: N = New World, O = Old World. Plio. = Pliocene; Quat. = Quaternary. Tip symbols: filled circles “⬤” denote transcriptomes, filled triangles “▲” denote genome skimming sequences, and unfilled stars “☆” denote herbarium or old silica-dried specimens. Tips without symbols come from living specimens of the Botanical Collection at Oberhof.

# LITERATURE CITED

**Frosch W, Cribb P**. **2012**. *Hardy Cypripedium: Species, hybrids and cultivation*. Kew Publishing Kew.

**Pease JB, Brown JW, Walker JF, Hinchliff CE, Smith SA**. **2018**. Quartet Sampling distinguishes lack of support from conflicting support in the green plant tree of life. *American journal of botany* **105**: 385–403.
