# Supplementary material for: Phylogenomic analysis of target enrichment and transcriptome data uncovers rapid radiation and extensive hybridization in the slipper orchid genus Cypripedium
Source: Ann Bot. 2024 Sep 12;134(7):1229–50. doi: 10.1093/aob/mcae161 (PMC11688532; doi:10.1093/aob/mcae161)
Supplement: mcae161_suppl_Supplementary_Materials_S1 [file mcae161_suppl_supplementary_materials_s1.docx]

*Library Preparation, Target Enrichment, and Sequencing*

**Methods S1**: Detailed library preparation and target enrichment protocol.

Total genomic DNA was isolated using the NucleoSpin Plant II kit: Genomic DNA from plants (Macherey-Nagel, Düren, Germany), following a modified version of the manufacturer's manual (Supplementary Data Table S5). Next, we quantified the concentration of all DNA samples using a Qubit 4 fluorometer with a Broad Range (BR) or High Sensitivity (HS) assay kit (Thermo Fisher Scientific, Waltham, Massachusetts, USA). After shearing the DNA to an average fragment size of 350 bp with a Covaris M220 Focused-ultrasonicator (Covaris, Woburn, Massachusetts, USA), we assessed the DNA fragment size distribution using a High Sensitivity DNA ScreenTape on a 4150 TapeStation System (Agilent Technologies, Santa Clara, California, USA).

For the preparation of the dual indexed libraries with the NEBNext Ultra II DNA Library Prep Kit for Illumina and the NEBNext Multiplex Oligos for Illumina (Dual Index Primers Set 1, New England Biolabs, Ipswich, Massachusetts, USA), we followed the recommended conditions of bead-based size selection according to distribution of DNA fragments per sample. We amplified the adaptor-ligated libraries in eight PCR cycles and measured DNA concentration with Qubit. The average fragment size of the libraries was assessed with the TapeStation. Prior to hybridization, the libraries were pooled in equal concentrations to include 250 ng of each library, with a maximum of 15 libraries per pooled library.

For the hybridization enrichment reaction, we combined the pooled libraries with the custom orchid-specific bait set Orchidaceae963 (Daicel Arbor Biosciences myBaits Target Capture Kit, Ann Arbor, MI, USA) and incubated at 60 °C (hybridization temperature, TH) for 16 hours overnight, following the Standard Protocol and the Blockers Mix setup designed for plants (myBaits Hybridization Capture for Targeted NGS, User Manual v. 5.02). The bead-based cleanup of the bait-target hybrids was performed at a wash temperature (TW) of 60 °C, and the hybridized libraries were subsequently amplified for 14 PCR cycles at 60 °C (annealing temperature, TA). Then, we purified the amplification reaction following the PCR clean-up protocol of the NucleoSpin Gel and PCR Clean-up kit (Macherey-Nagel, Düren, Germany). Finally, we checked the concentration and fragment size distribution of the libraries as before, using the Qubit and the TapeStation.

*Read Processing and Assembly*

**Methods S2**: Detailed read processing and assembly protocol for the target enrichment sequence data.

To create the set of orchid genome and transcriptome references, original target exon sequences from the Orchidaceae963 bait set (<https://github.com/laeserman/Orchidaceae963/blob/main/Orchidaceae963-targets.fa>) were concatenated into ‘genes’ and used to identify the corresponding complete CDS from the *Phalaenopsis equestris* genome using BLAST. When no hits were produced, we used the genome of *Dendrobium catenatum* instead. In the end, 950 out of the original 963 genes were extracted. Then, we used the raw transcriptome assembly from 17 orchids (Supplementary Data Table S4), consisting of *Vanilla shenzhenica* and 16 species of slipper orchids to extend the genome references. RNAseq data processing and transcriptome assembly followed Morales-Briones *et al.* (2021). We used CAPTUS v.0.9.90 (Ortiz *et al.*, 2023) to extract the corresponding loci from the 17 transcriptomes and the genomes of *Apostasia shenzhenica*, *D. catenatum*, *P. equestris*, and *Vanilla planifolia*. The extracted loci were used as the extended reference dataset for loci extraction in our own generated target enrichment data of *Cypripedium*.

We checked the quality of the raw reads using FastQC v.0.11.9 (Andrews, 2010) and MultiQC v.1.14 (Ewels *et al.,* 2016). PCR duplicates were removed with ParDRe v.2.1.5 (González-Domínguez and Schmidt, 2016). Then, using CAPTUS v.1.0.0 we trimmed the sequencing adaptors and low-quality bases, assembled the reads, and extracted the nuclear loci based on the reference dataset, setting the minimum contig depth to eight for the assembly step and the minimum percentages of identity and coverage to 75% and 50%, respectively, for the extraction step, to decrease the retention of contigs resulting from potential erroneous reads. As mentioned in the main text, the extracted loci were combined with loci extracted from the genomes and transcriptomes mentioned above, and the coding sequences of the combined dataset were extracted in FASTA files while keeping up to 25 paralog copies per sample.

*Orthology Inference of nuclear loci*

**Methods S3**: Detailed orthology inference protocol.

To infer orthologs for the phylogenetic reconstruction, we followed a modified version of the methods described in Morales-Briones *et al.* (2022; <https://bitbucket.org/dfmoralesb/target_enrichment_orthology>). First, we aligned each locus using the OMM_MACSE pipeline v.12.01 (Scornavacca *et al.*, 2019), which pre‐filters non‐homologous sequence fragments with HMMCleaner (Di Franco *et al.*, 2019), performs the multi-sequence alignment with MAFFT v.7.271, and carries out the translation accounting for frameshifts using MACSE v.2.08 (Ranwez *et al.,* 2018). OMM_MACSE was set to replace codons containing frameshifts with gaps. Next, we used Phyx (‘pxclsq’ Brown *et al.,* 2017) to remove aligned columns with more than 90% missing data. We inferred maximum likelihood (ML) homolog gene trees with IQ-TREE v.2.0.7 (Minh *et al.,* 2020) using extended model selection (Kalyaanamoorthy *et al.,* 2017) and no clade support. Then, we masked mono- and paraphyletic tips that belong to the same taxon, keeping the tips with the most unambiguous characters in the trimmed loci alignments for each taxon as described in (Yang and Smith, 2014). Spurious tips with unusually long branches were removed by reducing the tree diameter with TreeShrink v.1.3.9 (Mai and Mirarab, 2018). We ran TreeShrink with the ʻper‐gene’ mode, a false positive error rate threshold (α) of 0.05, and excluding the outgroups. We wrote FASTA files from the output homolog trees and followed the same steps as for the output FASTA files from CAPTUS, aligning them with OMM_MACSE and removing aligned columns with >90% missing data using Phyx. To infer the final homolog gene trees, we used IQ-TREE with extended model selection and assessed the clade support with 1,000 ultrafast bootstrap (BS) replicates. Following orthology inference using the tree-based “monophyletic outgroup” (MO) approach, as described in the main text, we wrote FASTA files from the output ortholog trees, re-aligned the loci using OMM_MACSE, and cleaned the alignments with Phyx, as before.

## *Testing for Hybridization Events*

**Methods S4**: Detailed methods regarding the phylogenetic network analyses for the test investigating intra-sectional hybridization within the subclades containing the three described hybrids that were included in this study, their putative parent taxa, and other taxa that share the same MRCA.

To test whether the hybrid status of the three described hybrids included in our taxon sampling is supported by our target enrichment data, we followed a similar approach to the one described for investigating potential hybridization at the backbone of the *Cypripedium* phylogeny in the main text. However, this time, we extracted the three subclades containing each hybrid, along with the putative parent taxa and other taxa sharing their MRCA. We reduced computational load by removing duplicated taxa, leaving a single representative for each monophyletic taxon. In the case of paraphyletic taxa, one representative taxon was left from each conspecific monophyletic subclade or from a group of consecutively diverging conspecific varieties. The number of maximum reticulation events and the number of optimal output networks were set to one and ten, respectively, for all three tests. The option “po” was specified to optimize the branch lengths and inheritance probabilities under full likelihood for the inferred *C. × alaskanum* networks, allowing for the direct comparison of all output networks with the Akaike’s Information Criterion (AIC), the corrected Akaike’s Information Criterion (AICc), and the Bayesian information criterion (BIC) scores calculated according to Yu *et al.* (2012). This optimization was only performed for the *C. × alaskanum* networks, which contain only four taxa, as it gets more time-consuming with an increasing number of taxa.

*Ancestral Range Estimation*

**Methods S5**: Division of nine areas for the biogeographical analyses in BioGeoBEARS.

The areas were divided based on the current distribution of the taxa included in the analysis, their proximity, and their distinct floristic and topoclimatic characteristics (e.g., climate, precipitation, elevation). South America (area A) was specified as a large distinct area since only a few outgroup slipper orchids are restricted to its Northern part [i.e., *Selenipedium aequinoctiale* and *Phragmipedium lindleyanum*; POWO, 2023]. We separated Central America and Mexico (area B, containing section Irapeana) from South America at the Isthmus of Panama and from North America at the deserts to its north (i.e., Baja, Mojave, Sonoran, and Chihuahuan deserts). North America was divided into three areas: the Northern area E (colder to polar, humid climate), the Eastern area D (lower altitudes, higher humidity and precipitation than the Western area), and the Western area C (higher altitudes, lower humidity and precipitation than Eastern area; Kottek *et al.*, 2006; Xiao *et al.*, 2020). All three areas match the distributions of different *Cypripedium* species, with only five species found in area E, while the Great Plains in the middle of North America seemingly create a distribution boundary for multiple *Cypripedium* species (Supplementary Data Fig. S1). The Mediterranean and Scandinavian regions were grouped with Western and Central Europe (area F) because only *C. calceolus* occurs in all three areas (Eccarius, 2009; Frosch and Cribb, 2012; Chen *et al.,* 2013; Walid *et al.,* 2019). We split area F from Eastern Europe and Russia (area G) at the boundaries of the Sarmatic and Pontic-South Siberian floristic provinces according to Schroeder (1998), as they have a more continental climate and match the limit of *C. guttatum’s* distribution in the European continent (Pfadenhauer and Klötzli, 2020; Supplementary Data Fig. S1). Area G was separated from the two Asiatic areas (namely, the Southeast Asian area I and the Northeast Asian area H) due to the higher number of unique *Cypripedium* species occurring there, as well as their different climates and floristic provinces (Kottek *et al.*, 2006; Fridley, 2008). The Southeast and Northeast Asian areas are split around the Qinling Mountains–Huaihe River Line (aka Qinling–Huaihe line), a natural topographic boundary that separates North temperate from South tropical China (Hu *et al.*, 2020), which also seems to create a boundary for the distribution of several *Cypripedium* species. To reduce the state space and thus the computational time of the analysis, Taiwan was included in the same area as Southeast Asia (area I), and Japan in the same area as Northeast Asia and Eastern Russia (area H) due to their proximity.

**Methods S6**: Input tree and taxon distribution information for the biogeographical analyses in BioGeoBEARS.

To prepare the input tree for the biogeographic analyses with BioGeoBEARS, we removed hybrids and duplicated species from the time-calibrated maximum clade credibility tree obtained from the divergence time estimation analysis. When multiple varieties were present, a single specimen per each accepted variety (according to Frosch and Cribb, 2012) was kept due to distinct distributions. *Cypripedium amesianum* and the ambiguous *C. macranthos* var. *alba* were also kept since they were not monophyletic with their presumably synonymous taxa [i.e., *C. yunnanense* and *C. macranthos* var. *albiflorum* (now synonym of *C. macranthos* var. *macranthos*), respectively], and, therefore, considered distinct taxonomic units for this analysis. We also removed non-slipper orchid taxa because of scarce sampling.

The taxon distributions were based on Eccarius (2009), Frosch and Cribb (2012), Chen *et al.* (2013), and Walid *et al.* (2019). The distributions of *C. amesianum* and *C. macranthos* var. *alba* were considered the same as their synonyms according to Frosch and Cribb (2012; Supplementary Data Figure S1).

# LITERATURE CITED

**Andrews S**. **2010**. FastQC: A Quality Control Tool for High Throughput Sequence Data [Online].

**Brown JW, Walker JF, Smith SA**. **2017**. Phyx: phylogenetic tools for unix. *Bioinformatics* **33**: 1886–1888.

**Chen SC, Liu ZJ, Chen LJ, Li LQ**. **2013**. *The Genus Cypripedium in China*. Peking: Science Press.

**Di Franco A, Poujol R, Baurain D, Philippe H**. **2019**. Evaluating the usefulness of alignment filtering methods to reduce the impact of errors on evolutionary inferences. *BMC Evolutionary Biology* **19**: 21.

**Eccarius W**. **2009**. *Orchideengattung Cypripedium*. EchinoMedia.

**Ewels P, Magnusson M, Lundin S, Käller M**. **2016**. MultiQC: summarize analysis results for multiple tools and samples in a single report. *Bioinformatics* **32**: 3047–3048.

**Fridley J**. **2008**. Of Asian Forests and European Fields: Eastern U.S. Plant Invasions in a Global Floristic Context. *PloS one* **3**: e3630.

**Frosch W, Cribb P**. **2012**. *Hardy Cypripedium: Species, hybrids and cultivation*. Kew Publishing Kew.

**González-Domínguez J, Schmidt B**. **2016**. ParDRe: faster parallel duplicated reads removal tool for sequencing studies. *Bioinformatics* **32**: 1562–1564.

**Hu Y, Yao Y, Kou Z**. **2020**. Exploring on the climate regionalization of Qinling-Daba mountains based on Geodetector-SVM model. *PLoS ONE* **15**: e0241047.

**Kalyaanamoorthy S, Minh BQ, Wong TKF, von Haeseler A, Jermiin LS**. **2017**. ModelFinder: fast model selection for accurate phylogenetic estimates. *Nature Methods* **14**: 587–589.

**Kottek M, Grieser J, Beck C, Rudolf B, Rubel F**. **2006**. World Map of the Köppen-Geiger Climate Classification Updated. *Meteorologische Zeitschrift* **15**: 259–263.

**Mai U, Mirarab S**. **2018**. TreeShrink: fast and accurate detection of outlier long branches in collections of phylogenetic trees. *BMC genomics* **19**: 23–40.

**Minh BQ, Schmidt HA, Chernomor O, *et al.*** **2020**. IQ-TREE 2: New Models and Efficient Methods for Phylogenetic Inference in the Genomic Era. *Molecular Biology and Evolution* **37**: 1530–1534.

**Morales-Briones DF, Kadereit G, Tefarikis DT, *et al.*** **2021**. Disentangling Sources of Gene Tree Discordance in Phylogenomic Data Sets: Testing Ancient Hybridizations in Amaranthaceae s.l. *Systematic Biology* **70**: 219–235.

**Morales-Briones DF, Gehrke B, Huang C-H, *et al.*** **2022**. Analysis of paralogs in target enrichment data pinpoints multiple ancient polyploidy events in Alchemilla sl (Rosaceae). *Systematic Biology* **71**: 190–207.

**Ortiz EM, Höwener A, Shigita G, *et al.*** **2023**. A novel phylogenomics pipeline reveals complex pattern of reticulate evolution in Cucurbitales. : 2023.10.27.564367.

**Pfadenhauer JS, Klötzli FA**. **2020**. Fundamentals towards Understanding Global Vegetation In: Pfadenhauer JS, Klötzli FA, eds. *Global Vegetation: Fundamentals, Ecology and Distribution*. Cham: Springer International Publishing, 1–120.

**POWO**. **2023**. *Plants of the World Online. Facilitated by the Royal Botanic Gardens, Kew. Published on the Internet.* http://www.plantsoftheworldonline.org/. 12 Feb. 2023.

**Ranwez V, Douzery EJ, Cambon C, Chantret N, Delsuc F**. **2018**. MACSE v2: toolkit for the alignment of coding sequences accounting for frameshifts and stop codons. *Molecular biology and evolution* **35**: 2582–2584.

**Schroeder FG**. **1998**. *Lehrbuch der Pflanzengeographie*. Quelle & Meyer.

**Scornavacca C, Belkhir K, Lopez J, *et al.*** **2019**. OrthoMaM v10: Scaling-Up Orthologous Coding Sequence and Exon Alignments with More than One Hundred Mammalian Genomes. *Molecular Biology and Evolution* **36**: 861–862.

**Walid N, Rebbas K, Krouchi F**. **2019**. Découverte de Cypripedium calceolus (Orchidaceae) au Djurdjura (Algérie), nouvelle pour l’Afrique du Nord. *Flora Mediterranea* **29**: 207–214.

**Xiao X, Liang S, He T, Wu D, Pei C, Gong J**. **2020**. *Estimating fractional snow cover from passive microwave brightness temperature data using MODIS snow cover product over North America*.

**Yang Y, Smith SA**. **2014**. Orthology inference in nonmodel organisms using transcriptomes and low-coverage genomes: improving accuracy and matrix occupancy for phylogenomics. *Molecular biology and evolution* **31**: 3081–3092.

**Yu Y, Degnan JH, Nakhleh L**. **2012**. The Probability of a Gene Tree Topology within a Phylogenetic Network with Applications to Hybridization Detection. *PLOS Genetics* **8**: e1002660.
