# Supplementary material for: Phylogenomic analysis of target enrichment and transcriptome data uncovers rapid radiation and extensive hybridization in the slipper orchid genus Cypripedium
Source: Ann Bot. 2024 Sep 12;134(7):1229–50. doi: 10.1093/aob/mcae161 (PMC11688532; doi:10.1093/aob/mcae161)
Supplement: mcae161_suppl_Supplementary_Materials_S2 [file mcae161_suppl_supplementary_materials_s2.docx]

## *Inferred Nuclear Species Phylogeny and Discordance*

**Results S1:** Species-level results of the *Cypripedium* phylogeny inferred based on target enrichment data of 913 nuclear loci.

Regarding the monophyly at the species level, two species within sect. *Cypripedium* that included numerous infraspecific taxa (i.e., varieties and one form), *C. macranthos* and *C. parviflorum*, were consistently recovered as paraphyletic in both phylogenies, with *C. franchetii* and *C. yunnanense* nested in the former, and *C. kentuckiense* nested in the latter. Two pairs of synonyms (according to Frosch and Cribb, 2012) formed monophyletic clades: that is, (*C. parviflorum* var. *makasin*, *C. parviflorum* var. *parviflorum*), and (*C. subtropicum*, *C. singchii*). However, the former pair was only formed between specimens from silica-gel material, while *C. parviflorum* specimens from herbarium material created a separate clade. In contrast, *C. amesianum* was more closely related to *C. froschii* rather than its synonymous species, *C. yunnanense*. Additionally, the ambiguous taxon *C. macranthos* var. *alba*, which was presumed to be either *C. macranthos* var. *albiflorum* (now a synonym of *C. macranthos* var. *macranthos*) or *C. macranthos* var. *album*, was more closely related to the equally white-flowered *C. macranthos* var. *rebunense*. Regarding the rest of the species, ASTRAL recovered most as monophyletic, grouping all conspecific specimens retrieved from different sources (i.e., Botanical Collection at Oberhof, herbarium M, and SRA), except the paraphyletic *C. calceolus* and *C. fargesii*. Notably, the three included hybrids following Frosch and Cribb (2012) were placed in the same clades as one or both of their putative parent taxa.

## *Hybridization Networks*

**Results S2:** Detailed phylogenetic network analysis results for the test investigating intra-sectional hybridization within the subclades containing the three described hybrids that were included in this study, their putative parent taxa, and other taxa that share the same MRCA.

To address our questions about whether the three known hybrid species included in this study are supported as products of hybridization between their putative parent taxa by our target enrichment data, we plotted the most likely phylogenetic networks that tested for one reticulation event. Regarding the test with *C. × alaskanum*, although the most likely network (Network 1, total log probability = ~ -556.90) indicated that *C. yatabeanum* is a hybrid between an unsampled taxon sharing an MRCA with sect. *Bifolia* (γ = 0.22) and *C. × alaskanum* (γ = 0.78), model selection comparing all networks resulting from this test suggested that the next three networks with the highest probability (total log probabilities for Networks 2: ~ -557.48, Network 3: ~ 557.64, and Network 4: ~ -557.92) were almost as good as the best model (deltaAIC, detlaAICc, and deltaBIC ≲ 2; Supplementary Data Fig. S15 and Table S10). Each of the latter networks provided support for different taxa being hybrids resulting from different crosses; therefore, it is not possible to infer accurate conclusions regarding the hybrid status of *C. × alaskanum* based on the PhyloNet analysis of our nuclear target enrichment data.

The most likely hybridization network of *C.* × *columbianum* (total log probability = ~ -16,825.20) supports that the clade containing the *C. parviflorum* varieties and *C. kentuckiense* was created following a hybridization between *C.* × *columbianum* and *C. candidum* (γ = 0.16 and 0.84, respectively; Supplementary Data Fig. S14 A). As for the network that includes *C.* × *ventricosum* (total log probability = ~ -72,700.35), the results indicate that both *C.* × *ventricosum* and *C. calceolus* are sister taxa that arose from a hybridization event between *C. macranthos* var. *rebunense* (γ = 0.38) and *C. shanxiense* (γ = 0.62; Supplementary Data Fig. S14 B). It is worth noting that when looking at the total log probabilities of all analyses testing for one to ten reticulation events in both subclades, the overall most likely models indicated a higher number of hybridizations; namely, 9 events in the *C.* × *columbianum* subclade (total log probability = ~ -72,263.36), and 10 events in the *C.* × *ventricosum* subclade (total log probability = ~ -16,778.53; Supplementary Data Fig. S13 and S16). However, since model selection was not performed for these analyses, further investigation is required to assess and validate these results.

# LITERATURE CITED

**Frosch W, Cribb P**. **2012**. *Hardy Cypripedium: Species, hybrids and cultivation*. Kew Publishing Kew.
