# Supplementary material for: Phylogenomic analysis of target enrichment and transcriptome data uncovers rapid radiation and extensive hybridization in the slipper orchid genus Cypripedium
Source: Ann Bot. 2024 Sep 12;134(7):1229–50. doi: 10.1093/aob/mcae161 (PMC11688532; doi:10.1093/aob/mcae161)
Supplement: mcae161_suppl_Supplementary_Materials_S3 [file mcae161_suppl_supplementary_materials_s3.docx]

## *Rapid Radiation and Hybridization Promoted Diversification*

**Discussion S1:** Detailed discussion of the phylogenetic network analysis results for the test investigating intra-sectional hybridization within the subclades containing the three described hybrids that were included in this study, their putative parent taxa, and other taxa that share the same MRCA.

Regarding reticulation at the intra-sectional level, our analyses indicated multiple potential reticulation events within sections *Bifolia*, *Cypripedium*, and *Subtropica*. Most nodes leading to the hybrids identified through our analyses had decreased concordance levels, including the MRCA node of the (*C. guttatum*, *C.* × *alaskanum*) clade. Although *C.* × *alaskanum* has been previously described as a natural hybrid between *C. yatabeanum* and *C. guttatum* by Brown (1995), it was difficult to assess its validity because no analysis accompanied the description (Cribb, 1997). Despite the hybrid status of *C.* × *alaskanum*, our target enrichment data suggested that *C. yatabeanum* is a hybrid between *C.* × *alaskanum* and an unsampled taxon closely related to the (*C. guttatum*, *C.* × *alaskanum*) clade. However, the support for this model was not significantly higher than that of other output networks, indicating different hybridization events. Therefore, additional analyses and molecular data are crucial to obtain robust molecular evidence for the hybrid status of *C.* × *alaskanum.*

As for the PhyloNet analyses within sect. *Cypripedium*, testing for a maximum of one hybridization event also yielded some unexpected results. Sheviak (1992) proposed that *C.* × *columbianum* is a hybrid between *C. montanum* and *C. parviflorum* var. *pubescens* based on an extensive survey and analysis of wild and herbarium specimens, with hybrids having intermediate morphological characteristics between the presumed parent taxa. The phylogenetic network containing *C.* × *columbianum* disagreed with Sheviak’s taxon description, identifying the *C. parviflorum* complex clade as a product of a hybrid cross between *C.* × *columbianum* and *C. candidum*, although their current ranges do not overlap in North America.

The results of a similar test on the dataset containing *C.* × *ventricosum* supported that the taxon did not constitute a hybrid from a cross between *C. calceolus* and *C. macranthos* as it was previously suggested based on the resemblance of its flower with an artificial hybrid between the presumed parent taxa (Rolfe, 1904, 1910; Cribb, 1997). Instead, the results indicated that *C.* × *ventricosum* and *C. calceolus* are products of a hybridization event between *C. shanxiense* and *C. macranthos.* Another hybrid, *C.* × *catherinae*, which occurs in Far East Russia (Siberia) and possibly in Korea and Northeast China, where the parent taxa’s distributions overlap, was already described from the hybridization between these two taxa (Frosch and Cribb, 2012; Chen *et al.*, 2013). *Cypripedium calceolus* and *C.* × *ventricosum* can also be found sympatrically with the parent taxa identified in our analysis, namely, in Siberia and Northeastern China, while *C. calceolus* is also found in Japan and on Sakhalin Island (Cribb, 1997; Frosch and Cribb, 2012; Chen *et al.*, 2013). Interestingly, *C.* × *ventricosum* and *C. calceolus* formed a clade with *C. shanxiense—*the putative parent taxon corresponding to the major edge (γ = 0.62)—in our target enrichment phylogenies, while in the chloroplast phylogeny, *C.* × *ventricosum* and one of the three *C. calceolus* specimens were more closely related to *C. macranthos* varieties. Moreover, a sister relationship is consistently recovered between *C. calceolus* and *C. shanxiense* (Fatihah *et al.*, 2011; Li *et al.*, 2011; Liu *et al.*, 2021; Szlachetko *et al.*, 2021) or between *C. calceolus* and *C. macranthos* in the absence of *C. shanxiense* (Cox *et al.,* 1997). Additionally, *C.* × *ventricosum* and *C. macranthos* formed a clade closely related to the (*C. calceolus*, *C. shanxiense*) clade in the phylogeny of Liu *et al.* (2021).

In support of Rolfe’s description, a statistical analysis of morphological and allozyme data from *C.* × *ventricosum* corroborated its status as a hybrid between *C. calceolus* and *C. macranthos* (Knyasev *et al.,* 2000). However, the fact that *C. calceolus* and *C.* × *ventricosum* may share the same parent taxa based on our results (i.e., *C. shanxiense* and *C. macranthos*) and that introgressive hybridization has been previously reported between *C. calceolus* and *C.* × *ventricosum* (Knyasev *et al.,* 2000) could elucidate the relationship between these species. Furthermore, it has been shown that the genetic structure of *C. shanxiense* was similar to *C. calceolus* from the eastern part of the range where they are sympatric, with the authors arguing that it may have been a result of introgressive hybridization, making the classification of these taxa even more challenging (Filippov and Andronova, 2011). At the same time, a hybrid has also been described between these two species (i.e., *C.* × *microsaccos*; Frosch and Cribb, 2012; Chen *et al.*, 2013).

When looking at the overall most probable phylogenetic networks for the subclades including *C.* × *ventricosum* and *C.* × *columbianum*, the results suggested that extensive hybridization events between sampled and unsampled taxa occurred within both subclades of sect. *Cypripedium*, which is supported by the evidence of pervasive hybridization within the genus, as mentioned before (Klier *et al.,* 1991; Hu *et al.,* 2011; Frosch and Cribb, 2012; Szlachetko *et al.,* 2017; Pupulin and Díaz-Morales, 2018). The mixed genetic and morphological signals created by these hybridizations could have significantly contributed to the difficulty in specific delimitation and taxonomic classification within the sect. *Cypripedium*, with the same taxa receiving a species, subspecies, variety, or hybrid rank by different taxonomists (e.g., *C. froschii*).

However, due to a high taxon sampling in most of our PhyloNet analyses except the one containing *C. × alaskanum*, we did not optimize the branch lengths and inheritance probabilities under full likelihood, as it becomes more computationally intensive and time-consuming with an increasing number of taxa. Consequently, we could not perform model selection to properly compare the network with the highest probability to other networks with lower probabilities inferred in these tests. Therefore, it is crucial to carry out additional studies and in-depth taxonomic revisions including further lines of evidence (e.g., a higher number and variety of molecular markers, allozyme markers, genetic studies at the population level, geometric morphometric studies of homologous morphological traits, etc.) to thoroughly assess the validity of the hybridization events that we identified in the genus *Cypripedium* through our investigation.

# LITERATURE CITED

**Brown PM**. **1995**. New taxa and taxonomic notes. **1**: 195–200.

**Chen SC, Liu ZJ, Chen LJ, Li LQ**. **2013**. *The Genus Cypripedium in China*. Peking: Science Press.

**Cox AV, Pridgeon AM, Albert VA, Chase MW**. **1997**. Phylogenetics of the slipper orchids (Cypripedioideae, Orchidaceae): Nuclear rDNA ITS sequences. *Plant Systematics and Evolution* **208**: 197–223.

**Cribb P**. **1997**. *The Genus Cypripedium*. Portland: Timber Press.

**Fatihah HN, Fay M, Maxted N**. **2011**. Molecular Phylogenetics of *Cypripedium* L. (Cypripedioideae: Orchidaceae) Based on Plastid and Nuclear DNA Sequences. *Journal of Agrobiotechnology* **2**: 111–118.

**Filippov E, Andronova E**. **2011**. Genetic differentiation in plants of the genus *Cypripedium* from Russia inferred from allozyme data. *Genetika* **47**: 615–23.

**Frosch W, Cribb P**. **2012**. *Hardy Cypripedium: Species, hybrids and cultivation*. Kew Publishing Kew.

**Hu S-J, Hu H, Yan N, Huang J-L, Li S-Y**. **2011**. Hybridization and asymmetric introgression between *Cypripedium tibeticum* and *C. yunnanense* in Shangrila County, Yunnan Province, China. *Nordic Journal of Botany* **29**: 625–631.

**Klier K, Leoschke MJ, Wendel JF**. **1991**. Hybridization and Introgression in White and Yellow Ladyslipper Orchids (*Cypripedium candidum* and *C. pubescens*). *Journal of Heredity* **82**: 305–318.

**Knyasev MS, Kulikov PV, Knyaseva OI, Semerikov VL**. **2000**. Interspecific hybridization in northern Eurasian *Cypripedium*: morphometric and genetic evidence of the hybrid origin of *C. ventricosum*. *Lindleyana* **15**: 10–20.

**Li J, Liu Z, Salazar GA, *et al.*** **2011**. Molecular phylogeny of *Cypripedium* (Orchidaceae: Cypripedioideae) inferred from multiple nuclear and chloroplast regions. *Molecular Phylogenetics and Evolution* **61**: 308–320.

**Liu H, Jacquemyn H, Chen W, *et al.*** **2021**. Niche evolution and historical biogeography of lady slipper orchids in North America and Eurasia. *Journal of Biogeography* **48**: 2727–2741.

**Pupulin F, Díaz-Morales M**. **2018**. On the meaning of *Cypripedium × grande* (Orchidaceae) and its taxonomic history, with a new name for the nothospecies occurring in Costa Rica and Panama. *Phytotaxa* **382**: 167.

**Rolfe RA**. **1904**. *Cypripedium calceolus × macranthos*. **12**: 185.

**Rolfe RA**. **1910**. *Cypripedium × ventricosum*. **18**: 215.

**Sheviak C**. **1992**. Natural hybridisation between *Cypripedium montanum* and its yellow-lipped relatives. **61**: 558.

**Szlachetko DL, Kolanowska M, Muller F, Vannini J, Rojek J, Górniak M**. **2017**. First Guatemalan record of natural hybridisation between Neotropical species of the lady’s slipper orchid (Orchidaceae, Cypripedioideae). *PeerJ* **5**: e4162.

**Szlachetko DL, Górniak M, Kowalkowska AK, Kolanowska M, Jurczak-Kurek A, Archila Morales F**. **2021**. The natural history of the genus *Cypripedium* (Orchidaceae). *Plant Biosystems-An International Journal Dealing with all Aspects of Plant Biology* **155**: 772–796.
